# Kainate receptor activation induces glycine receptor endocytosis through PKC deSUMOylation

**DOI:** 10.1038/ncomms5980

**Published:** 2014-09-19

**Authors:** Hao Sun, Li Lu, Yong Zuo, Yan Wang, Yingfu Jiao, Wei-Zheng Zeng, Chao Huang, Michael X. Zhu, Gerald W. Zamponi, Tong Zhou, Tian-Le Xu, Jinke Cheng, Yong Li

**Affiliations:** 1 Department of Biochemistry and Molecular Cell Biology, Shanghai Key Laboratory for Tumor Microenvironment and Inflammation, Institute of Medical Sciences, Shanghai Jiao Tong University School of Medicine, Shanghai 200025, China; 2 Center for Translational Medicine, Shanghai Chest Hospital, Shanghai Jiao Tong University School of Medicine, Shanghai 200025, China; 3 Department of Integrative Biology and Pharmacology, The University of Texas Health Science Center at Houston, Houston, Texas 77030, USA; 4 Department of Physiology and Pharmacology, Hotchkiss Brain Institute, The University of Calgary, Calgary T2N 4 N1, Alberta, Canada; 5 Department of Medicine, The University of Alabama at Birmingham, Birmingham, Alabama 35294, USA

## Abstract

Surface expression and regulated endocytosis of glycine receptors (GlyRs) play a critical function in balancing neuronal excitability. SUMOylation (SUMO modification) is of critical importance for maintaining neuronal function in the central nervous system. Here we show that activation of kainate receptors (KARs) causes GlyR endocytosis in a calcium- and protein kinase C (PKC)-dependent manner, leading to reduced GlyR-mediated synaptic activity in cultured spinal cord neurons and the superficial dorsal horn of rat spinal cord slices. This effect requires SUMO1/sentrin-specific peptidase 1 (SENP1)-mediated deSUMOylation of PKC, indicating that the crosstalk between KARs and GlyRs relies on the SUMOylation status of PKC. SENP1-mediated deSUMOylation of PKC is involved in the kainate-induced GlyR endocytosis and thus plays an important role in the anti-homeostatic regulation between excitatory and inhibitory ligand-gated ion channels. Altogether, we have identified a SUMOylation-dependent regulatory pathway for GlyR endocytosis, which may have important physiological implications for proper neuronal excitability.

## Abstract (web summary)

Maintenance of proper membrane excitability is vital to neuronal function and in several neuronal types this relies on a balance between receptor-mediated excitation and inhibition. Here the authors report a crosstalk between excitatory kainate receptors and inhibitory glycine receptors that relies on the SUMOylation status of PKC.

## Graphical abstract

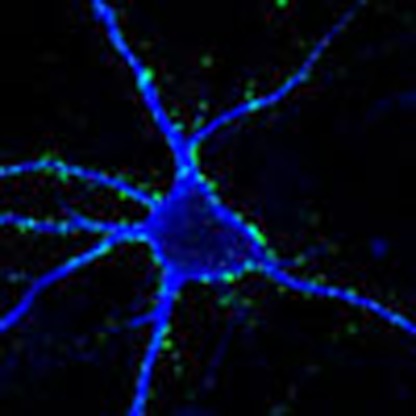

It is well established that maintenance of proper membrane excitability is vital to neuronal function and primarily relies on a finely tuned balance between receptor-mediated excitation and inhibition^1^. In neurons of the brainstem and spinal cord, this balance is primarily controlled by ionotropic glutamate receptors and glycine receptors (GlyRs)^2^,^3^,^4^. Kainate receptors (KARs), a subtype of ionotropic glutamate receptors, regulate synaptic transmission and neuronal excitability by acting at pre-, post- and extrasynaptic sites, depending on their specific localization and density^5^,^6^. The inhibitory GlyRs, on the other hand, are ligand-gated chloride channels that mediate fast synaptic inhibition in spinal cord and brainstem^7^,^8^. GlyRs consist of α1–4 and β subunits that can form homomeric (5α subunits) or heteromeric complexes with 3α and 2β subunits^9^. They are localized at either glycinergic^10^,^11^,^12^,^13^ or mixed glycinergic/GABAergic (gamma-aminobutyric acid) postsynaptic sites^13^,^14^,^15^.

There are several ways to regulate the number of GlyRs at glycinergic synapses to fine-tune synaptic efficacy, including lateral movement of the receptors from and to the postsynaptic loci and receptor stabilization at postsynaptic specializations, as well as adjusting the relative rates of receptor endocytosis and exocytosis^16^,^17^. It has been shown that activation of *N*-methyl-D-aspartate receptors (NMDARs) induced a decrease in lateral diffusion of GlyRs in a manner that required calcium influx and calcium release from internal stores and this decrease was accompanied with an increase in GlyR cluster number and glycinergic miniature inhibitory postsynaptic current (mIPSC) amplitude^18^. Activation of protein kinase C (PKC) modulates GlyRs function and regulates endocytosis of α1 homomeric GlyRs in a dynamin-dependent manner^9^,^19^. Phosphorylation by PKC at residue S403 of the GlyR β-loop diminishes the affinity between GlyRs and gephyrin scaffolds, resulting in an increased GlyR diffusion and consequently decreased GlyR levels at inhibitory synapses^20^. Because NMDA or kainate stimulation has also been shown to cause a PKC-dependent internalization of GluK2-containing KARs, which trafficked to early endosomes and then were sorted into either recycling or degradative pathways^5^, we investigated whether and how PKC exerts a role in the functional interaction between GlyRs and KARs.

Interestingly, the PKC-dependent internalization of GluK2 has been shown to require SUMOylation (where SUMO is small ubiquitin-like modifier) of the KARs^21^,^22^. SUMOylation is a post-translation modification that modifies protein function by covalently binding a member of the SUMO family to the target protein, and it has been shown to play crucial roles in the central nervous system (CNS) including regulation of ion-channel function^23^,^24^ and membrane protein endocytosis, thus controlling synaptic functions^25^. The levels of SUMOylation in neurons can be dynamically modulated by the activity-dependent redistribution of the SUMOylation machinery at pre- and postsynaptic sites, which consequently regulates complex physiological processes^26^. SUMOylation is readily and rapidly reversed by the isopeptidase activity of SUMO/sentrin-specific proteases (SENPs). The SUMOylation state of a specific protein is determined by the balance between SUMOylation and SENP-mediated deconjugation, and as such the mechanisms that regulate these processes are of particular interest to unravel the roles of SUMOylation in the CNS.

Here we report a novel anti-homeostatic type of regulation between GlyRs and KARs that involves KAR activation-induced internalization of GlyRs, as seen by a reduced GlyR cell surface density, in a manner that is dependent on SENP1-mediated PKC deSUMOylation. This crosstalk between KARs and GlyRs may serve to maintain the proper neuronal excitability and to regulate excitatory–inhibitory balance in the CNS.

## Results

### KAR activation leads to GlyR internalization

**Figure 1:**
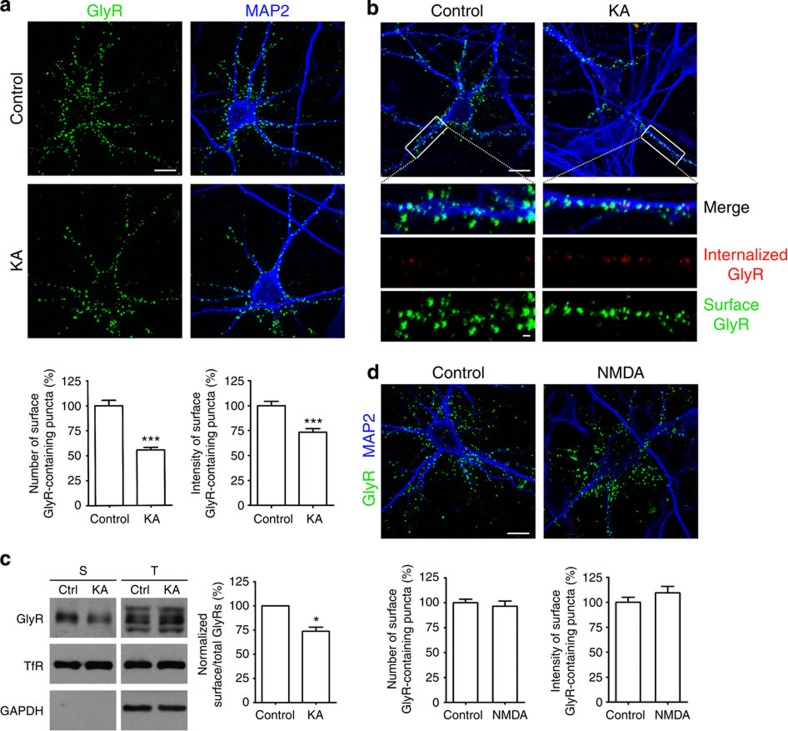
Kainate induces GlyR internalization in spinal cord neurons. (**a**) Antibody-feeding assay under non-permeabilized conditions labelled cell surface GlyRs (green) in cultured rat spinal cord neurons untreated (Control) or briefly treated with kainate (KA, 200 μM, 1 min). Neurons were then permeabilized and labelled with MAP2 (blue) to show cell body and processes. Puncta numbers and integrated fluorescence intensities of surface GlyRs from all images analysed by ImageJ and values are normalized to untreated controls. Data are means±s.e.m. from at least three experiments; the total numbers of neurons analysed (*n*) ranged from 66 to 71 cells per condition. ***P<0.001 compared with control by Student’s *t*-test. Scale bar, 10 μm. (**b**) Endocytosis of GlyRs was visualized by the antibody-feeding immunofluorescence assay between untreated (Control) or briefly treated with kainate (KA) for 1 min. Pre-labelled GlyRs remaining on the surface are in green; internalized receptors are in red. Magnified view of the region enclosed by the white square in the lower panel. Scale bar, 10 μm (original, upper) and 1 μm (magnified, lower). (**c**) Surface biotinylation of GlyRs in cultured rat spinal cord neurons untreated (control) or treated with kainate (KA, 1 min). Endogenous transferrin receptor (TfR) is shown as a surface protein control and endogenous glyceraldehyde 3-phosphate dehydrogenase as a cytoplasmic protein control. S, surface; T, total; Ctrl, control. KA treatment decreased surface GlyRs to 73.6±4.3% (means±s.e.m.) of the untreated control. *P<0.05 compared with untreated control by Student’s paired *t*-test *P*=0.0258, *n*=3. (**d**) NMDA treatment (30 μM, 1 min) did not evoke significant change in the number and intensity of surface GlyRs as quantified by the antibody-feeding assay under non-permeabilized conditions. Data are shown as in **d** with the bar graphs representing the means±s.e.m. from at least three experiments; the total numbers of neurons analysed (*n*) ranged from 28 to 38 cells per condition. Scale bar, 10 μm.

KARs modulate neurotransmitter release at the presynaptic terminal and contribute to fast excitatory synaptic transmission at the postsynaptic membrane^27^,^28^,^29^,^30^,^31^,^32^,^33^,^34^. Cell surface density/stability of GlyRs is dynamically regulated in a PKC-dependent manner by receptor endocytosis and exocytosis^16^,^17^. Given that KAR activation can trigger PKC signalling^5^,^21^,^25^,^35^, we hypothesized that KARs may alter the dynamics of GlyR trafficking. To test this possibility, cell surface GlyRs were labelled using antibodies raised against either amino acids 1–10 of the GlyR α1 subunit or the extracellular amino termini of GlyRs on cultured live spinal cord neurons under non-permeabilized conditions. Under control conditions, surface fluorescent immunoreactivity of GlyRs exhibited a punctate distribution over the neuronal dendrites and soma (Fig. 1a). A brief treatment with kainate induced a marked decrease in the number of puncta and fluorescence intensity of surface GlyRs (Fig. 1a). The effect of kainate was dose dependent, being detectable at as low as 0.5 μM and approaching the maximum at around 10 μM (Supplementary Fig. 1a). Visualizing the internalized GlyRs under permeabilized conditions in an antibody-feeding immunofluorescence assay also revealed increases in the number and intensity of GlyR-positive labels in the spinal cord neuron dendrites in response to kainate treatment (Fig. 1b), and the kainate-induced loss of GlyRs from the cell surface was further confirmed by the spinal cord neuron surface biotinylation experiment (Fig. 1c). On the other hand, co-staining with a presynaptic marker glycine transporter 2 showed no obvious loss of synapses (Supplementary Fig. 1c), indicating that the loss of surface GlyRs was solely due to receptor internalization but not a decrease in the number of synapses. In contrast, treatment with NMDA or α-amino-3-hydroxy-5-methyl-4-isoxazolepropionic acid (AMPA) did not significantly change the surface number or intensity of GlyRs (Fig. 1d; Supplementary Fig. 1b), suggesting a specific regulation of surface GlyRs by kainate.

### Kainate-induced GlyR endocytosis is mediated predominantly by KARs

It is well known that kainate acts as an agonist at both AMPA receptors (AMPARs) and KARs^5^,^33^,^36^,^37^,^38^. Several lines of evidence suggest that the decrease in surface GlyRs was mediated predominantly by KARs rather than AMPARs.

**Figure 2:**
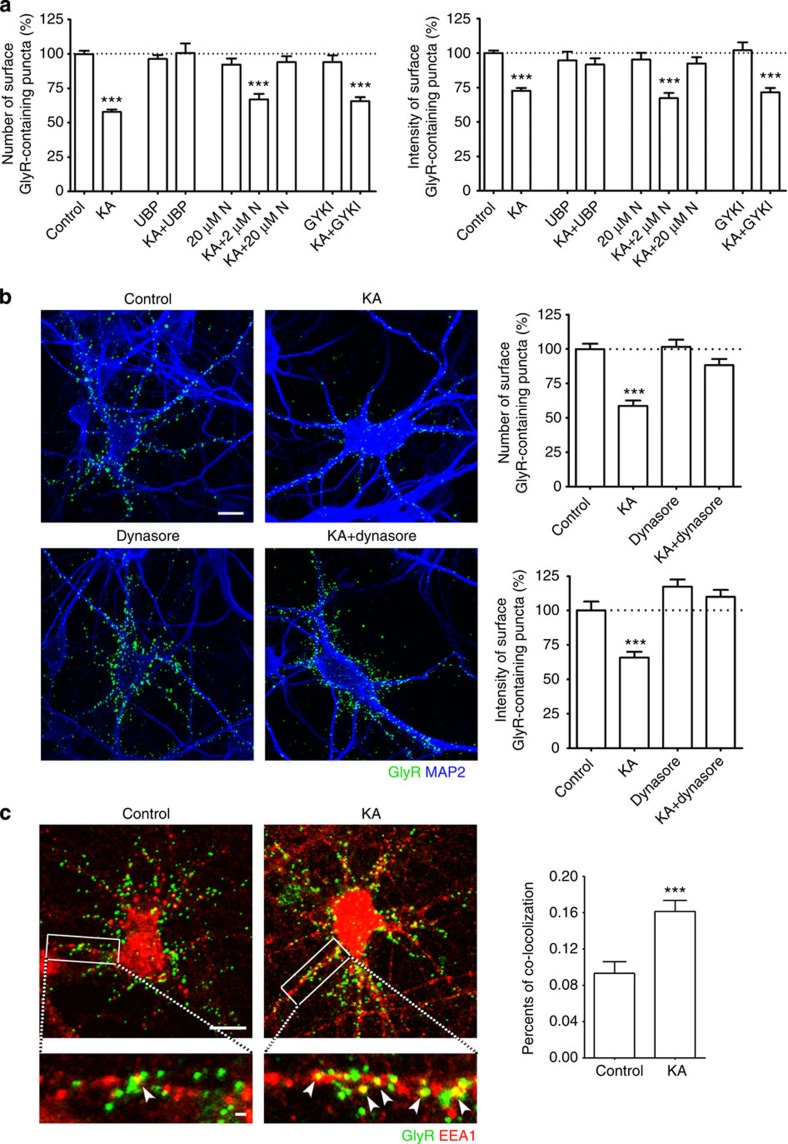
Kainate-induced surface GlyR decrease occurs via dynamin-dependent endocytic pathway. (**a**) Effect of pre-incubation with antagonists for KARs and/or AMPA receptors (UBP (UBP301) 50 μM, N (NBQX) 2 and 20 μM and GYKI (GYKI53655) 10 μM) on kainate (KA)-induced GlyR internalization. Data are normalized to control and shown as means±s.e.m. from at least three experiments; the total numbers of neurons analysed (*n*) ranged from 20 to 72 cells per condition. (**b**) Inhibition of kainate (KA) effects on GlyR surface expression by dynasore (80 μM, 1 h). Puncta numbers (upper, bar graph) and integrated fluorescence intensities (lower, bar graph) of surface GlyRs from all images analysed by ImageJ and values normalized to untreated controls (GlyR—green, MAP2—blue). Data are means±s.e.m. from four experiments; the total numbers of neurons analysed (*n*) ranged from 20 to 43 cells per condition. Scale bar, 10 μm. (**c**) Overlap between internalized GlyRs and early endosome marker EEA1. Internalized and remaining pre-labelled surface GlyR receptors are shown in green and the EEA1 is shown in red. Magnified view of the region enclosed by the white square in the lower panel. The arrowheads indicate the colocalization of internalized GlyR with EEA1 (yellow). The bar graph shows quantification of means±s.e.m. from three experiments using the colocalization finder plugin of ImageJ software; the total numbers of neurons analysed (*n*) were 11 for both Control and KA. Scale bar, 10 μm (original, upper) and 1 μm (magnified, lower). ***P<0.001 compared with control, by one-way analysis of variance with pairwise comparison by Tukey’s *post hoc* test.

First, we tested a number of pharmacological blockers. As shown in Fig. 2a, the kainate-induced decrease in surface GlyRs was not observed in the presence of either the KAR antagonist UBP301 (50 μM) or a high concentration of NBQX (2,3-dihydroxy-6-nitro-7-sulfamoyl-benzo[f]quinoxaline-2,3-dione) (20 μM) that blocks both AMPA and KA receptors. Incubation of UBP301 and NBQX under resting conditions excluded the possibility that either UBP301 or NBQX alone had any effect on the GlyR surface expression. In contrast, pre-incubation of cells with lower concentrations of GYKI53655 (10 μM) or NBQX (2 μM), at which they selectively blocked AMPARs over KARs, did not prevent the effects of kainate with regard to both the number and intensity of surface GlyRs. These data demonstrated that the decrease in surface GlyRs was completely abolished when KARs were inhibited but not when AMPARs were selectively inhibited. Second, as stated above, treatment with AMPA did not induce an appreciable decrease in surface GlyRs (Supplementary Fig. 1b). Taken together, these pharmacological profiles support the idea that the decrease in surface GlyRs by kainate treatment was mediated predominantly by KARs.

### Internalized GlyRs are sorted into early endosomes

It has been reported that excitatory transmission led to more dynamic clustering of GlyRs at postsynaptic sites by lateral diffusion^18^. To determine whether the observed kainate effects on GlyRs occurred via endocytosis or lateral diffusion, we took advantage of the cell-permeable dynamin inhibitor dynasore, a blocker of endocytosis. Pre-incubation with dynasore resulted in partial blockage of the kainate-induced decrease in GlyR cell surface levels (Fig. 2b). Treatment of neurons with sucrose (a known blocker of endocytosis) also attenuated the effect of kainate on GlyR internalization (Supplementary Fig. 2). These findings support the idea that kainate-induced decrease in the density of surface GlyRs is mediated by dynamin-dependent receptor endocytosis.

Once a receptor cargo has been internalized, it enters the endocytic pathway. To further determine whether the internalized GlyRs were delivered to intracellular endocytic compartments, immunofluorescence staining was performed on cultured spinal neurons to visualize the colocalization between internalized GlyRs and an early endosome marker, EEA1. Under basal conditions, few GlyRs were colocalized with EEA1, suggesting weak constitutive internalization of GlyRs. A brief application of kainate significantly increased the overlap between internalized GlyRs and EEA1 (Fig. 2c). Together, these data indicate that GlyRs undergo constitutive endocytosis under basal conditions and more newly internalized GlyRs are sorted to somatodendritic early endosomes on kainate stimulation.

### Kainate-mediated GlyR endocytosis is activity- and Ca^2+^-dependent

**Figure 3:**
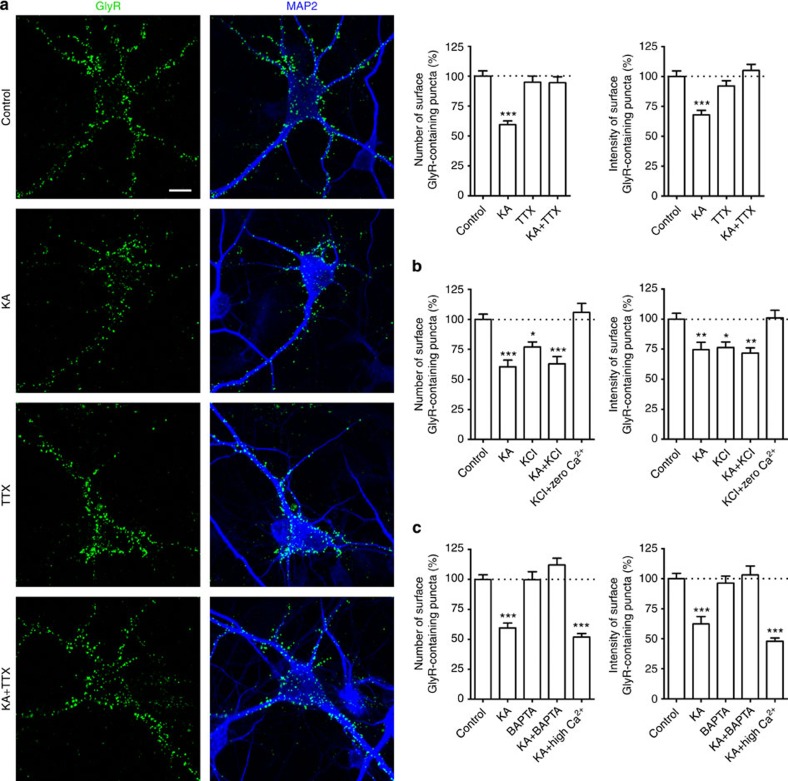
Kainate induces GlyR internalization in an activity- and calcium-dependent manner. (**a**) Surface GlyRs assessed by the antibody-feeding assay under non-permeabilized conditions from control and kainate (KA)-treated neurons with or without pretreatment with tetrodotoxin (TTX, 1 μM, 1 h). Data show quantification of surface GlyR cluster numbers and intensities in means±s.e.m. from four experiments; the total numbers of neurons analysed (*n*) ranged from 35 to 55 cells per condition. Scale bar, 10 μm. (**b**) Effects of stimulating cultured neurons with high KCl on surface GlyRs. Incubating neurons with KCl (50 mM, 1 min) induced GlyR internalization similarly as kainate (KA), but the effect was not seen in the extracellular calcium-free medium. Data show quantification of surface GlyR cluster numbers and intensities in means±s.e.m. from three experiments; the total numbers of neurons analysed (*n*) ranged from 14 to 30 cells per condition. (**c**) Cultured neurons were either pre-incubated with BAPTA-AM (50 μM, 1 h) or placed in a medium that contained 8 mM Ca^2+^ when stimulated with KA. Quantification was performed as in Fig. 1a and data are shown as means±s.e.m. of surface GlyR cluster numbers and intensities from three experiments; the total numbers of neurons analysed (*n*) ranged from 21 to 60 cells per condition. *P<0.05, **P<0.01, ***P<0.001 compared with the respective control, by one-way analysis of variance with pairwise comparison by Tukey’s *post hoc* test.

To test whether the endocytosis of GlyRs is dependent on neuronal activities, we measured GlyRs internalization in cultured spinal cord neurons in the presence of tetrodotoxin (TTX) or in response to high KCl treatment. Surprisingly, pre-incubation of live neurons with the voltage-gated Na^+^ channel blocker TTX impaired the subsequent GlyRs endocytosis in response to the brief kainate treatment (Fig. 3a). It has been shown that suppressing synaptic activity using TTX decreases the deSUMOylating enzyme SENP1 and consequently alters the status of protein SUMOylation^39^ (see Discussion). These data suggest that spontaneous neuronal activity in the culture was necessary for the kainate-induced GlyR internalization. In contrast, high KCl treatment alone, which is known to raise intracellular calcium concentrations, promoted GlyR endocytosis (Fig. 3b). Altogether, these data suggest that GlyRs undergo activity-dependent endocytosis.

It is well established that KARs are permeable to Ca^2+^ and that Ca^2+^ influx via KARs is pivotal for various forms of activity-dependent changes in synaptic efficacy. Thus, we examined whether Ca^2+^ influx via KARs was necessary for KAR-mediated GlyR endocytosis. First, bath application of the membrane-permeable Ca^2+^ chelator BAPTA-AM (1,2-Bis(2-aminophenoxy)ethane-*N*,*N*,*N*′,*N*′-tetraacetic acid tetrakis(acetoxymethyl ester), a cell-permeable derivative that is widely used as an intracellular calcium sponge) impaired kainate-induced GlyR endocytosis, resulting in retention of GlyRs on the cell surface (Fig. 3c) after kainate stimulation. By contrast, when the extracellular Ca^2+^ concentration was elevated to 8 mM (high Ca^2+^), kainate stimulation caused a more robust decrease in surface GlyRs (Fig. 3c). Second, either chelating extracellular calcium with EGTA or omitting calcium from extracellular solution (nominally calcium free) blocked the kainate-induced GlyR endocytosis (Supplementary Fig. 3a), indicating that the GlyR endocytosis induced by kainate was dependent on calcium influx. Third, high KCl-induced GlyR endocytosis was largely abolished in the calcium-free extracellular solution (Fig. 3b), suggesting that KCl-induced GlyR endocytosis was mediated by calcium influx, likely via voltage-gated calcium channels (VGCCs) opened in response to membrane depolarization. On the other hand, although kainate-induced GlyR endocytosis was largely inhibited by various KAR antagonists such as UBP301 and NBQX, it was not significantly attenuated by the application of an L-type calcium channel blocker, nifedipine (Fig. 2a; Supplementary Fig. 3b). These results indicate that calcium influx, either through KARs or VGCCs, can cause GlyR endocytosis and that the KAR-dependent regulation of GlyR endocytosis requires intracellular calcium elevation.

### Roles of PKC and PKA on kainate-induced GlyR endocytosis

**Figure 4:**
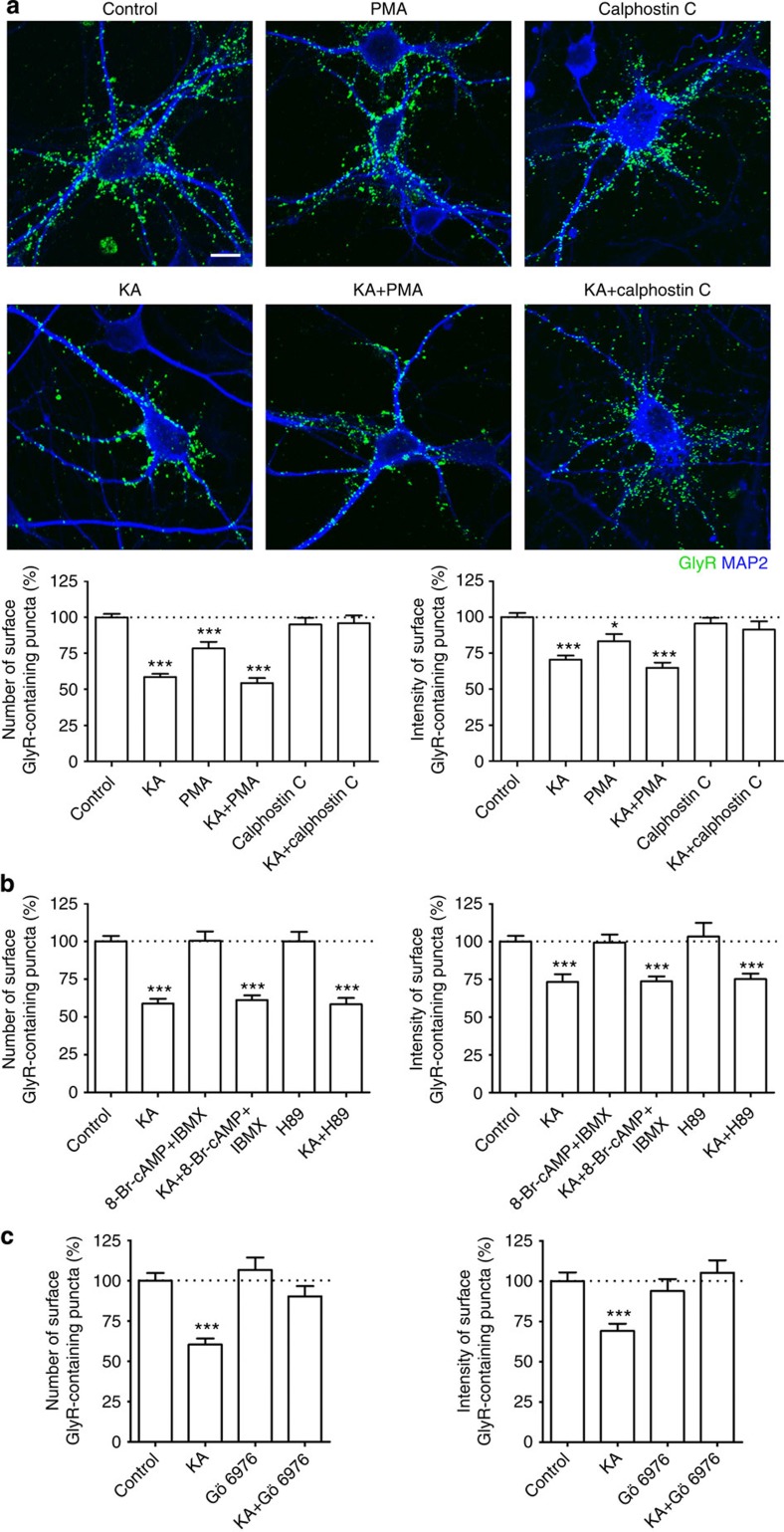
Kainate induces GlyR internalization through activation of PKC. (**a**) Effect of PKC activator (PMA, 1 μM, 1 h) or PKC inhibitor (calphostin C, 1 μM, 1 h) alone or in combination with kainate (KA) on the endocytosis of GlyRs. Surface GlyRs assessed by the antibody-feeding assay under non-permeabilized conditions in neurons treated with drugs as indicated and quantification obtained as in Fig. 1a. Data are means±s.e.m. from four experiments; the total numbers of neurons analysed (*n*) ranged from 29 to 55 cells per condition. Scale bar, 10 μm. (**b**) Histograms depict the effects of PKA activator (8-Br-cAMP 100 μM plus IBMX 100 μM, 1 h) and inhibitor (H89, 10 μM, 1 h) on the puncta number (left) and intensity (right) of surface GlyRs following kainate (KA) stimulation. Data are normalized to untreated controls and shown as means±s.e.m. from three experiments; the total numbers of neurons analysed (*n*) ranged from 14 to 40 cells per condition. (**c**) Bar graph showing the effect of Gö6976 (0.5 μM, 1 h) on puncta number (left) and intensity (right) of surface GlyRs following kainate (KA) treatment. Data are normalized to untreated controls and shown as means±s.e.m. from at least two experiments; the total numbers of neurons analysed (*n*) ranged from 10 to 25 cells per condition. *P<0.05, ***P<0.001 compared with control, by one-way analysis of variance with pairwise comparison by Tukey’s *post hoc* test.

Direct phosphorylation of receptor subunits regulates endocytosis of glutamate receptors, including that of AMPARs, NMDARs and KARs^6^,^21^,^22^,^40^,^41^,^42^. It has recently been shown that diffusion properties of GlyRs and the interaction between GlyRs and gephyrin were regulated through PKC phosphorylation of the GlyR β-subunit^20^. To test whether protein phosphorylation was involved in kainate-evoked GlyR endocytosis, we examined the effects of selective activators and inhibitors of PKA (protein kinase A) and PKC on kainate-induced GlyR endocytosis. Although the expressed GluK2 subunits in mammalian cells was directly phosphorylated and modulated by PKA^43^,^44^, neither the PKA activator 8-Br-cAMP (8-bromo cyclic AMP) plus IBMX (3-isobutyl-1-methylxanthine) nor the PKA inhibitor H89 affected kainate-evoked GlyR endocytosis in cultured spinal cord neurons (Fig. 4b). These results indicate that PKA plays no role in kainate-evoked GlyR internalization. In contrast, inhibitors of PKC calphostin C or Gö6976 (selectively inhibits Ca^2+^-dependent isoforms of PKC) had remarkable effects on kainate-evoked GlyR endocytosis. Treatment with calphostin C (Fig. 4a) or Gö6976 (Fig. 4c) nearly abolished all endocytosis, whereas activation of PKC by phorbol ester (PMA) had no further effect on the kainate-induced GlyR internalization (Fig. 4a). Interestingly, PMA alone also significantly increased GlyR internalization (Fig. 4a), indicating that PKC may directly regulate GlyR endocytosis under the present experimental conditions. Altogether, our data suggest that PKC activation is required for kainate-evoked GlyR internalization.

### SENP1 deficiency impairs kainate-induced GlyR endocytosis

**Figure 5:**
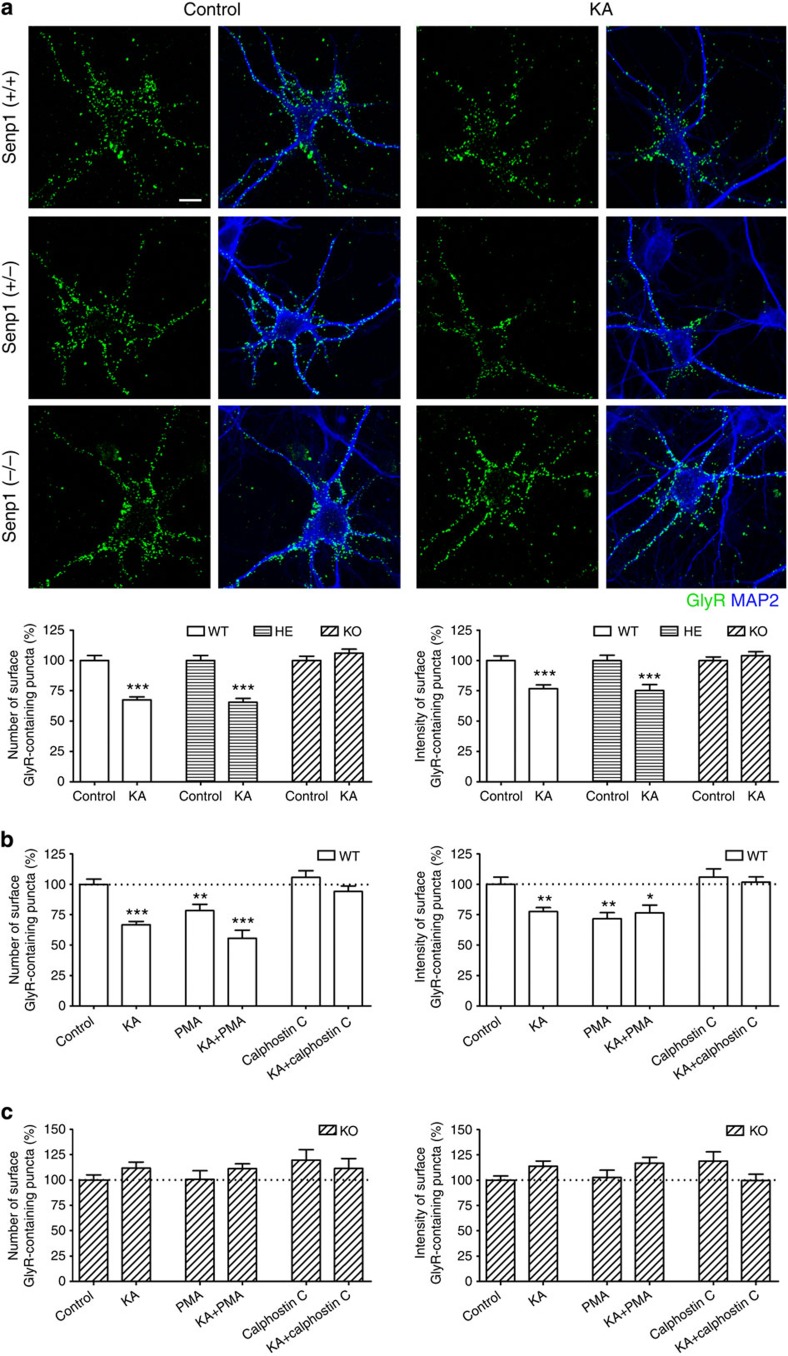
SENP1 deficiency impairs the kainate/PKC-induced GlyR endocytosis. (**a**) Surface GlyRs assessed by the antibody-feeding assay under non-permeabilized conditions in neurons prepared from *SENP1*^+/+^ (WT), *SENP1*^+/−^ (HE, heterozygous) and *SENP1*^−/−^ (KO) mice. Quantification data are means±s.e.m. from at least six experiments; the total numbers of neurons analysed (*n*) ranged from 56 to 84 cells per condition. Scale bar, 10 μm. (**b**,**c**) Quantification of surface GlyRs in *SENP1*^+/+^ (**b**) and *SENP*^−/−^ (**c**) neurons treated with PMA or calphostin C alone or in combination with kainate (KA) as indicated in Fig. 4a. Data are means±s.e.m. from three to four experiments; the total numbers of neurons analysed (*n*) ranged from 13 to 39 cells per condition. *P<0.05, **P<0.01, ***P<0.001 compared with the respective control, by one-way analysis of variance with pairwise comparison by Tukey’s *post hoc* test.

SUMOylation and deSUMOylation by SUMO-specific E1, E2, E3 and SENPs have been shown to regulate a large number of biological processes and membrane protein functions^45^,^46^,^47^. SENP1 is a protease that removes SUMO from conjugated targets (isopeptidase activity) and generates mature SUMO from SUMO precursors (endopeptidase activity)^46^,^48^,^49^. To explore the possible involvement of SUMOylation in GlyR endocytosis, we measured surface GlyR levels on SENP1-mutant spinal cord neurons with and without kainate treatment. As shown in Fig. 5a, kainate failed to decrease the number and intensity of surface GlyR on neurons prepared from SENP1 knockout (*SENP1*^−/−^) mouse embryos, whereas the downregulation of surface GlyRs by kainate was clearly detectable in wild-type (*SENP1*^+/+^) and SENP1 heterozygous (*SENP1*^+/−^) neurons. Furthermore, overexpression of SENP1 in *SENP1*^−/−^ neurons restored kainate-induced GlyR endocytosis almost to the *SENP1*^+/+^ levels (Supplementary Fig. 4f; Fig. 5a). These findings suggest that SENP1-mediated deSUMOylation is required for the kainate-induced GlyR endocytosis.

To determine whether the reduced GlyR internalization in *SENP1*^−/−^ neurons was mediated indirectly by changes in surface density of KARs and/or calcium influx via KARs, due to their own internalization on kainate stimulation^25^, we first compared the number and intensity of surface GlyRs (Supplementary Fig. 4a) as well as the intensity of GluK2-containing KARs (Supplementary Fig. 4b) on spinal cord neurons prepared from *SENP1*^+/+^, *SENP1*^+/−^ and *SENP1*^−/−^ embryos and found no change in these values among the three genotypes. Second, the amplitudes of kainate-activated currents (*I*_KA_) were similar among neurons from the three genotypes (Supplementary Fig. 4c). These results suggested that the effect of SUMOylation on kainate-induced GlyR endocytosis was unrelated to any SUMOylation-elicited change in KAR expression and function. Third, we compared kainate-induced increases in intracellular calcium levels by calcium imaging of spinal cord neurons prepared from *SENP1*^+/−^ and *SENP1*^−/−^ embryos. However, no significant difference in the calcium response to kainate treatment was found between these neurons (Supplementary Fig. 4d), indicating that the effect of *SENP1*^−/−^ on GlyR internalization likely occurred downstream of calcium entry via KARs. This suggested that either GlyRs were directly regulated by SUMOylation or, alternatively, SUMOylation modulated the molecular machinery involved in the internalization process of GlyRs. Finally, to determine whether GlyRs might be directly SUMOylated, we examined GlyR SUMOylation in HEK293T cells transiently co-transfected with Flag-SUMO1 and GlyR α1 or α2 subunit plasmids. However, as shown in Supplementary Fig. 4e, GlyRs do not appear to be a substrate for SUMOylation. These observations suggested that neither GluK2-containing KARs nor GlyRs were likely to be at the root of the SENP1 effects on kainate-induced GlyR internalization in spinal cord neurons.

### SENP1 regulates the status of PKC SUMOylation

Surprisingly, PMA alone failed to affect GlyR surface expression levels in spinal cord neurons from *SENP1*^−/−^ mouse embryos (Fig. 5c), whereas it reduced surface GlyRs on neurons from *SENP1*^+/+^ neurons (Fig. 5b). We therefore hypothesized that SENP1 may control GlyR endocytosis by altering the SUMOylation status of PKC.

**Figure 6:**
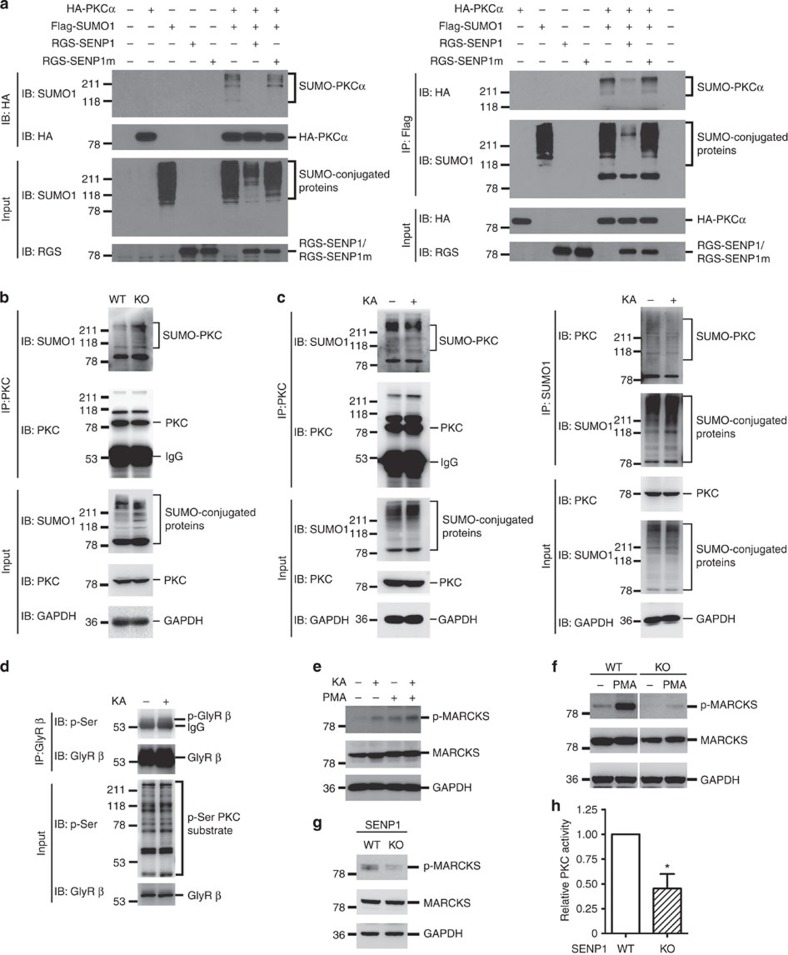
SENP1 regulates SUMOylation of PKC and changes phosphorylation of PKC substrate. (**a**) SENP1 deSUMOylates PKC *in vivo*. CHO-K1 cells were transfected with HA-tagged PKC, Flag-tagged SUMO1, RGS-tagged SENP1 or its catalytically inactive SENP1m as indicated. Cell lysates were precipitated with anti-HA antibody (left) or anti-Flag antibody (right; IP) and analysed by immunoblotting using anti-SUMO1, HA (IB). Whole-cell lysates were immunoblotted (IB) with anti-SUMO1, HA and RGS antibodies as indicated. (**b**) The SUMOylated form of PKC was determined by immunoprecipitation with anti-PKC antibodies followed by western blotting with anti-SUMO1 antibodies in *SENP1*^+/+^ (WT) and *SENP1*^−/−^ (KO) MEF cells. (**c**) Neurons were untreated (−) or treated (+) with kainate (KA, 200 μM, 1 min) and immunoprecipitated using anti-PKC antibodies (left panel) or anti-SUMO1 antibodies (right panel), followed by immunoblotting using anti-SUMO1 or anti-PKC antibodies as indicated. (**d**) Kainate enhanced phosphorylation of GlyR β subunit by PKC. Cultured spinal cord neurons were untreated (−) or treated (+) with kainate (KA, 200 μM, 1 min) and cell lysates subject to immunoprecipitation using anti-GlyR β subunit antibody, followed by immunoblotting using anti-phospho-(Ser) PKC substrate (upper panel) or anti-GlyR β subunit (lower panel) antibodies, respectively, as indicated. Note: GlyR β subunit-specific bands were around 58 kDa, in part overlapping with the IgG heavy chain either in upper or lower panels. Data are representative of at least three independent experiments. (**e**–**g**) Total and phosphorylated MARCKS were determined by immunoblotting in spinal cord neurons treated with kainate (KA, 200 μM, 1 min), PMA (1 μM, 1 h) or both as indicated (**e**), *SENP1*^+/+^ (WT) and *SENP1*^−/−^ (KO) MEF cells treated with either PMA or not as indicated (**f**) and tissue homogenates from E13.5 *SENP1*^+/+^ (WT) and *SENP1*^−/−^ (KO) embryos (**g**). Glyceraldehyde 3-phosphate dehydrogenase was used as a loading control. (**h**) PKC activity was determined by enzyme-linked immunosorbent assay-based relative kinase assays in tissue homogenates from E13.5 *SENP1*^*+/+*^ (WT) and *SENP1*^−/−^ (KO) embryos and was measured using the PKC kinase activity kit (Enzo) according to the manufacturer’s instructions. Data are means±s.e.m. from three experiments. *P<0.05 compared with WT by Student’s *t*-test *P*=0.0334.

To test this hypothesis, we first examined whether classical PKCs could be modified by SUMOylation in CHO-K1 cells transiently transfected with Flag-SUMO1 and HA-PKCα plasmids (Fig. 6a). Immunoblotting using anti-SUMO1 antibodies revealed multiple SUMO-conjugate bands in anti-HA (for PKC) immunoprecipitates from cells co-transfected with Flag-SUMO1 and HA-PKC but not in those transfected with Flag-SUMO1 or HA-PKC alone (Fig. 6a, left panels). The approximate sizes of these bands are consistent with that estimated for PKC plus the addition of two or more ~20 kDa SUMO proteins, presumably representing modifications of PKC either at multiple sites by SUMO1, or at a single site with the formation of SUMO1 chains. Cell lysates were immunoprecipitated using anti-Flag (to immunoprecipitate SUMOylated proteins) antibodies, followed by immunoblotting using anti-HA (to visualize SUMOylated HA-PKC) antibodies. The results again indicated the presence of a series of slowly migrating bands in cells that coexpressed HA-PKC and Flag-SUMO1, confirming direct SUMOylation of PKC (Fig. 6a, right panels). Consistent with these higher molecular weight species representing SUMO modification of PKC, sequence analysis using SUMOplot (Abgent) identified multiple putative SUMOylation sites in classical PKCs.

To test whether SENP1 directly deSUMOylates PKC, HA-PKCα and Flag-SUMO1 were coexpressed with RGS-His-tagged SENP1 or a catalytically inactive mutant SENP1 (SENP1m) in CHO-K1 cells. As expected, overexpression of SENP1, but not SENP1m, completely abrogated SUMOylated PKC (Fig. 6a). These results indicate that SENP1 acts as the deSUMOylating enzyme for PKC. In addition, SUMOylation of PKCα was also measured under denaturing conditions after transiently transfecting Flag-SUMO1 and HA-PKCα in CHO-K1 cells. Cells were lysed in a buffer containing 1% SDS and subject to immunoprecipitation using anti-HA antibodies. This was followed by immunoblotting using either anti-SUMO1 or anti-HA (Supplementary Fig. 6a, left panels) antibodies. Conversely, the lysates were immunoprecipitated using anti-Flag antibodies and the precipitants subject to immunoblotting using anti-HA or anti-SUMO1 antibodies (Supplementary Fig. 6a, right panels). The results indicated that the SUMOylation of PKCα was preserved under denaturing conditions (Supplementary Fig. 6a).

Furthermore, we examined whether a SUMOylated form of PKC could be detected in SENP1 knockout mouse embryonic fibroblast (MEF) cells. The SUMOylated form of PKC was increased in *SENP1*^−/−^ MEF cells, as shown by immunoprecipitation with anti-PKC followed by western blotting with anti-SUMO1 (Fig. 6b). We have also repeated this experiment under denaturing conditions in tissue homogenates from *SENP1*^*+/+*^and *SENP1*^−/−^ embryos (instead of MEF cells). The results obtained (Supplementary Fig. 6b) are similar to that under non-denaturing conditions (Fig. 6b). In addition, to detect the endogenous SUMOylated PKCs and to verify whether they were modulated by kainate stimulation in spinal cord neurons, cell lysates from cultured neurons were immunoprecipitated using either anti-PKC antibodies (Fig. 6c, left panels) or anti-SUMO1 antibodies (Fig. 6c, right panels), followed by immunoblotting using either anti-SUMO1 or anti-PKC antibodies, respectively. From both assays, SUMOylated PKCs were detected and the amount reduced with kainate stimulation, demonstrating that the SUMOylation status of PKC was regulated by KAR activation. Similar results were obtained under denaturing conditions (Supplementary Fig. 6c). To further confirm that PKC SUMOylation was decreased by kainate treatment in neurons using a different set of antibodies, we tested the SUMOylation of HA-PKCα by Flag-SUMO1 in co-transfected spinal cord neurons without and with kainate treatment. As expected, HA-PKCα SUMOylation was decreased on kainate treatment (Supplementary Fig. 6d). These observations suggest that PKC is modified by SUMO1 and kainate treatment reduces PKC SUMOylation in spinal cord neurons.

In addition, myristoylated alanine-rich C kinase substrate (MARCKS) is a major cellular substrate of PKC commonly utilized to evaluate PKC activity^50^. As shown in Fig. 6e, both KA and PMA significantly increased MARCKS phosphorylation in cultured spinal cord neurons, supporting the idea that KA stimulation increased PKC activity. The enhanced PKC activity by KA might phosphorylate GlyRs and in turn cause receptor internalization. Previously, it was reported that S403 of the GlyR β subunit was mainly responsible for changes in GlyR dynamics^20^. The phosphorylation status of GlyR β subunit, therefore, could represent an estimate of PKC activity on GlyRs. Indeed, the phosphorylation level of GlyR β subunit was enhanced on kainate treatment (Fig. 6d), further supporting kainate-induced GlyR internalization by enhancing PKC phosphorylation of the GlyRs. To examine the effect of PKC SUMOylation status on PKC activity, we measured MARCKS phosphorylation levels in the absence and presence of PMA treatment in MEF cells from *SENP1*^+/+^ and *SENP1*^−/−^ mice. Although MARCKS phosphorylation was increased following PMA treatment in both cell groups, the increase was much more pronounced in *SENP1*^+/+^ than in *SENP1*^−/−^ MEF cells (Fig. 6f). Consistent with this idea, MARCKS phosphorylation was decreased in tissue homogenates from *SENP1*^−/−^ embryos (Fig. 6g), indicating that the status of PKC SUMOylation affects the PKC activity. To strengthen this point, we also compared the PKC activity in tissue homogenates from *SENP1*^*+/+*^and *SENP1*^−/−^ embryos using a PKC kinase activity kit. *SENP1*^−/−^ exhibited about 50% less PKC activity as compared with *SENP1*^+/+^, suggesting that enhanced SUMOylation of PKC inhibited its activity (Fig. 6h).

**Figure 7:**
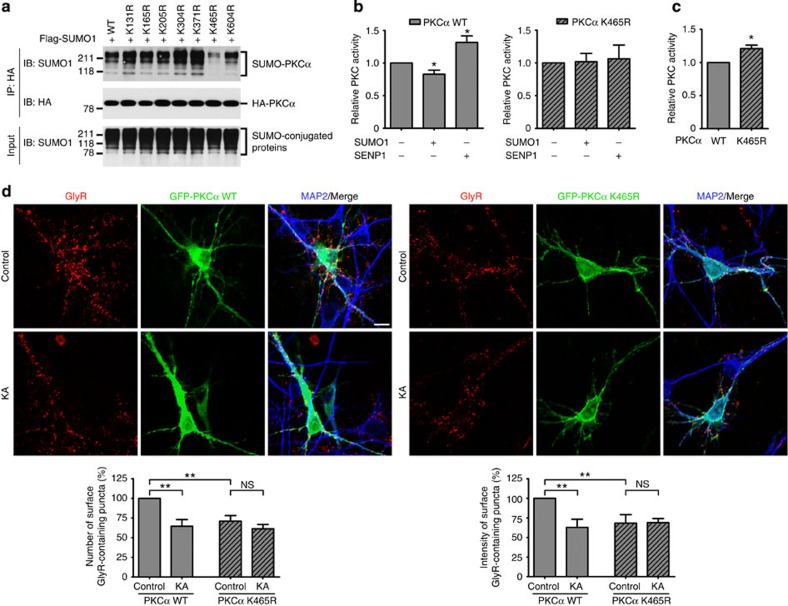
Effect of SENP1-mediated PKC deSUMOylation on PKC activity. (**a**) Mapping potential SUMOylation sites on PKCα. CHO-K1 cells were either transfected with wild-type PKCα or its lysine-to-arginine mutants as indicated. All PKCα constructs were tagged with an HA epitope and Flag-tagged SUMO1 was included as indicated. Cells were lysed and immunoprecipitated using anti-HA antibodies, followed by immunoblotting with anti-SUMO1 antibodies. (**b**) SUMOylation and deSUMOylation of wild-type PKCα (PKCα-WT) affect its activity, but not K465R PKCα mutant (PKCα-K465R). CHO-K1 cells were transfected with HA-PKCα or HA-PKCα K465R either together with Flag-SUMO1 or with SENP1 as indicated. Cell lysates were incubated with anti-HA agarose beads (Pierce) at 4 °C for 3 h to immunoprecipitate HA-tagged PKCα or PKCα K465R. PKC enzyme-linked immunosorbent assay-based relative kinase assays were performed on HA-tagged fractions. Data are means±s.e.m. from three independent experiments and plotted as relative kinase activity compared with respective control. (**c**) PKCα K465R increases its relative kinase activity. PKC activity was analysed in the same *in vitro* assay as in **b**. Data are means±s.e.m. from four independent experiments and plotted as relative kinase activity. *P<0.05 compared with WT by Student’s *t*-test *P*=0.0282. (**d**) PKCα K465R enhances GlyR internalization in non-stimulated conditions. Cultured spinal cord neurons transiently transfected with the K465R mutant and wild type of PKCα either in non-stimulated conditions or with KA treatment. Surface GlyRs evaluated by the antibody-feeding assay under non-permeabilized conditions in cultured spinal cord neurons overexpressed K465R and wild type of PKCα. Quantification data are means±s.e.m. from three experiments; the total numbers of neurons analysed (*n*) nine cells per condition. *P<0.05, **P<0.01, NS: no significant difference by Student’s *t*-test. Scale bar, 10 μm.

To determine the primary site(s) of PKCα SUMOylation, we generated single mutants bearing lysine-to-arginine substitutions at seven putative SUMOylation sites: K131, K165, K205, K304, K371, K465 and K604R, using site-directed mutagenesis. The results shown in Fig. 7a demonstrate that the K465R substitution greatly reduced the degree of PKCα SUMOylation, suggesting that K465 is the primary, although not the only, site of PKCα SUMOylation. Moreover, our data showed that PKC activity of the K465R mutant was unaffected by SUMOylation (Fig. 7b). By contrast, SUMOylation of wild-type PKCα inhibited its activity either *in vivo* or *in vitro* (Figs 6f–h and 7b). Furthermore, we examined whether the K465R substitution altered its PKC activity in the same *in vitro* assay and observed that K465R mutant was significantly more active than the wild-type PKC (Fig. 7c). In addition, we also evaluated the effect of the K465R substitution on GlyR function. To this aim, we assessed kainate-induced GlyR endocytosis in cultured spinal cord neurons transiently transfected with the K465R mutant of PKCα and found that overexpression of PKCα K465 mutant also attenuated the effect of kainate on GlyR endocytosis (Fig. 7d; Supplementary Fig. 7). In addition, under non-stimulated conditions, the surface GlyR level in cultured neurons that overexpressed the K465R mutant was significantly lower than in those that overexpressed wild-type PKCα (Fig. 7d), indicating that the mutant mimicked the SENP1-mediated deSUMOylated state that was normally induced by kainate stimulation in spinal cord neurons. Taken together, these findings support the mechanism by which SENP1 regulates kainate-induced GlyR endocytosis via changing the SUMOylation status and activity of PKC.

### KAR activation depresses GlyR-mediated synaptic activity

**Figure 8:**
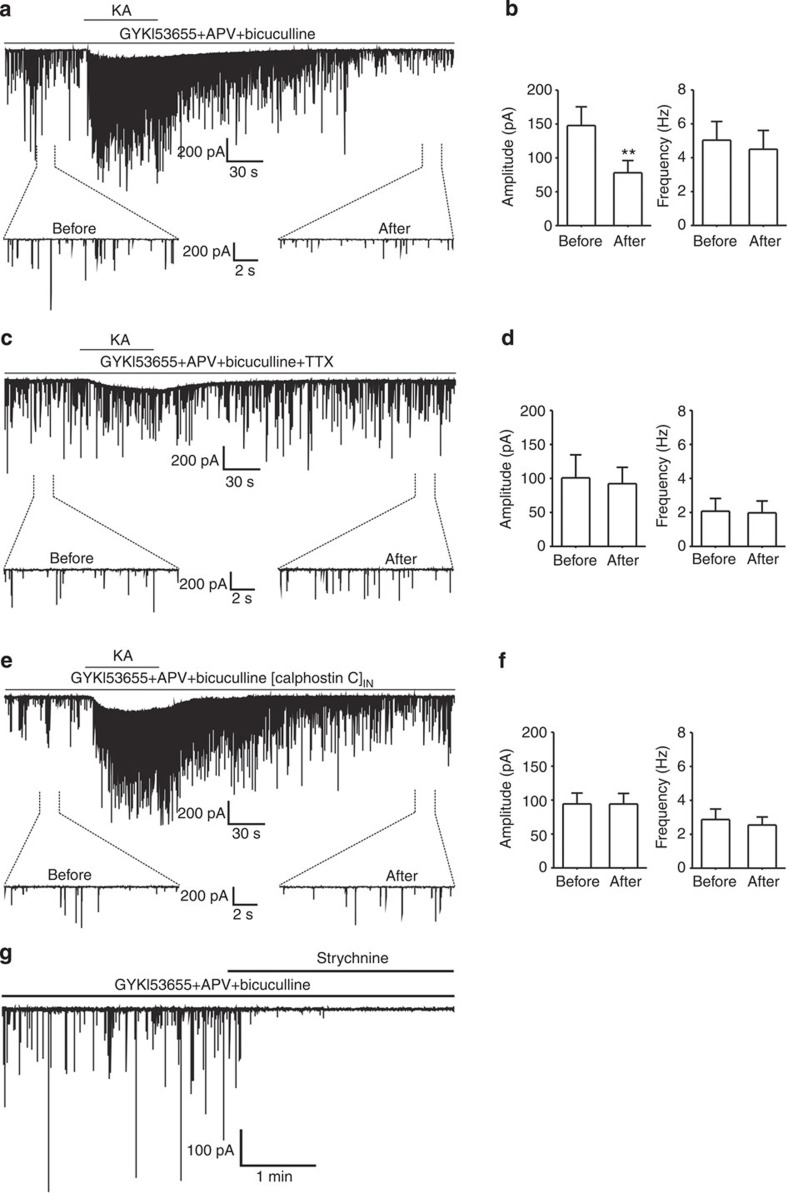
Kainate decreases glycinergic sIPSCs. (**a**) Whole-cell patch-clamp recordings on neurons of the superficial dorsal horn of rat spinal cord slices were performed at a holding potential of −70 mV. Representative traces are shown for before kainate (KA) treatment (before) and 3 min after 1 min KA treatment (after). Glycinergic sIPSCs were isolated by addition of 10 μM bicuculline, 10 μM GYKI53655 and 50 μM APV (D-(-)-2-Amino-5-phosphonopentanoic acid). (**b**) Quantifications of the sIPSC amplitude (left) and frequency (right). Data are means±s.e.m. from seven neurons. **P<0.01 compared with before (before KA), by Student’s *t*-test. (**c**) Representative records showing glycinergic mIPSCs, recorded in the presence of TTX (1 μM), before and after the kainate (KA) treatment. (**d**) Average values of the mIPSCs amplitude (left) and frequency (right) in the presence of TTX. Data are means±s.e.m. from five neurons. (**e**) Inhibition of PKC by calphostin C (1 μM) applied through the recording pipette prevented the inhibitory action of kainate (KA) on glycinergic sIPSCs. (**f**) Quantifications of the sIPSC amplitude (left) and frequency (right) in the presense of calphostin C. Data are means±s.e.m. from eight neurons. (**g**) Representative trace showing that glycinergic sIPSC events were completely blocked by 1 μM strychnine.

To understand the physiological significance of this observation *in vivo*, glycinergic spontaneous IPSCs (sIPSCs) were studied in the superficial dorsal horn of rat spinal cord slices by whole-cell recordings. A high symmetrical Cl^−^ concentration was used in these electrophysiological experiments and glycinergic sIPSCs were recorded at −70 mV in the presence of antagonists for GABA_A_ receptors (bicuculline), AMPARs (GYKI53655) and NMDARs (D-AP5). This allowed us to cleanly isolate glycinergic currents, as evidenced by their complete elimination on strychnine treatment (Fig. 8g). On 1 min kainate treatment, the average amplitude of glycinergic sIPSCs decreased from 147.7±27.7 pA in control conditions to 78.0±18.0 pA after kainate treatment (*n*=7, *P*=0.0043, by paired *t*-test), whereas the frequency of glycinergic sIPSCs did not change significantly (Fig. 8a,b; from 5.0±1.1 to 4.5±1.1 Hz, *n*=7, *P*=0.3854, by paired *t*-test).

In accord with the finding that kainate-induced GlyR endocytosis was dependent on neuronal activities, in the presence of 1 μM TTX, 50 μM D-AP5, 10 μM GYKI53655 and 10 μM bicuculline, neither the mean amplitude nor the frequency of glycinergic mIPSCs was significantly altered by kainate treatment on the superficial dorsal horn of rat spinal cord slices (amplitude: before kainate, 100.8±23.9 pA; after kainate, 92.2±24.0 pA, *n*=5, *P*=0.6795, by paired *t*-test; frequency: before kainate, 2.1±0.8 Hz; after kainate, 2.0±0.7 Hz, *n*=5, *P*=0.7134, by paired *t*-test; Fig. 8c,d), although TTX selectively blocked larger amplitude events and significantly decreased the mean amplitude and frequency of glycinergic IPSCs.

In addition, to investigate the effect of PKC on glycinergic sIPSCs, we delivered calphostin C via the recording pipette to the recorded neurons. As shown in Fig. 8e,f, neither the frequency nor the amplitude of glycinergic sIPSCs was changed by the kainate treatment with calphostin C in the pipette (amplitude: before kainate, 94.2±16.1 pA; after kainate, 94.1±16.0 pA, *n*=8, *P*=0.9861, by paired *t*-test; frequency: before kainate, 2.9±0.6 Hz; after kainate, 2.5±0.5 Hz, *n*=8, *P*=0.2379, by paired *t*-test). This contrasts the inhibitory effect of kainate treatment on the amplitude of glycinergic sIPSCs in the absence of the PKC inhibitor (Fig. 8a,b), demonstrating a critical role of PKC in mediating KAR-dependent regulation of GlyRs density in the postsynaptic membrane. There was no change in access resistance throughout the experiment, thus supporting the specificity of the effects. Altogether, our data show that activation of KARs affects glycinergic synaptic transmission, likely by virtue of regulating GlyRs density in the postsynaptic membrane.

## Discussion

The foregoing results elucidate the molecular mechanisms underlying the crosstalk between excitatory and inhibitory neurotransmission in rodent spinal cord neurons involving glutamatergic KARs and glycinergic GlyRs. Activation of KARs leads to inhibition of postsynaptic GlyR function by promoting GlyRs internalization via a pathway that involves SENP1-mediated PKC deSUMOylation. Both calcium entry and PKC activation are required to mediate kainate-evoked endocytosis of GlyRs, as reflected by decreases in both the number and fluorescence intensity of extracellularly labelled GlyR clusters in immunocytochemical staining of cultured neurons. The kainate- as well as PMA-induced GlyR endocytosis is absent in spinal cord neurons prepared from *SENP1*^−/−^ mice, suggesting a crucial role of SENP1-mediated deSUMOylation in this regulatory process. Functional and biochemical experiments ruled out the change in the surface expression or function of KARs by SENP1 knockout or covalent SUMO binding to GlyRs as the primary reason for SUMOylation-mediated GlyR endocytosis. However, PKC SUMOylation in spinal cord neurons was reduced following kainate treatment, suggesting PKC as a target of the SENP1 regulation. Consistent with the image data and biochemical analysis, electrophysiological recordings in the superficial dorsal horn of rat spinal cord slices demonstrated that the amplitude, but not frequency, of glycinergic sIPSCs was reduced by kainate treatment and inhibiting PKC abolished such an effect. Our data thus demonstrate an important role of SENP1-mediated PKC deSUMOylation in the crosstalk in which activation of excitatory KARs induces downregulation of inhibitory GlyRs via PKC-dependent receptor internalization, revealing a previously unknown role for SUMO in the regulation of normal synaptic function.

A previous study has revealed that surface levels of homomeric α1GlyRs are regulated by dynamin-dependent endocytosis^19^. Using dynasore and sucrose, we confirmed that endogenous GlyRs in spinal cord neurons were endocytosed into cytoplasmic compartments in response to a brief kainate treatment (Fig. 2b; Supplementary Fig. 2). Further supporting this conclusion, we found that the colocalization between internalized GlyRs and EEA1 increased following kainate treatment (Fig. 2c). The kainate-induced GlyR endocytosis reduces the number of postsynaptic GlyRs, thus explaining why kainate reduced the amplitude of glycinergic sIPSCs recorded in neurons from spinal cord slices (Fig. 8a,b).

It is intriguing that both intracellular Ca^2+^ rise and PKC activation are involved in the kainate-induced GlyR endocytosis. Previous studies have shown either enhancement of GlyR currents by intracellular Ca^2+^ elevations^51^,^52^ or by PKC activators^53^,^54^,^55^ or reduction of glycine-evoked responses by PKC activators^56^,^57^. These seemingly contradicting results were not well explained, but many factors, including the cell type-specific expression and differential assembly of different GlyR subunits as well as multiple roles of PKC isoforms, might contribute to the observed differential effects of PKC activators on GlyRs^9^. Recently, it was reported that phosphorylation of residue S403 at the GlyR β-loop by PKC diminished the association between GlyRs and gephyrin scaffolds, resulting in increased GlyR diffusion and consequently a decrease in the GlyR levels at inhibitory synapses^20^, and our results show that the phosphorylation level of GlyR β subunit was increased by kainate treatment (Fig. 6d). This would fit well with previous reports showing that GlyR internalization was facilitated by PKC activation^3^,^19^ and our current results that PKC is required for the crosstalk between KARs and GlyRs leads to GlyR internalization. In this context, our finding that SUMOylation status of PKC is critical in mediating GlyR endocytosis reveals an additional level of PKC regulation in controlling synaptic function.

As classical PKC isoforms are activated by Ca^2+^, it is likely that the PKC-dependent regulation of GlyR internalization occurs in response to intracellular Ca^2+^ increases. Indeed, we show that kainate-induced GlyR endocytosis was abolished by removing extracellular Ca^2+^ or chelating intracellular Ca^2+^ by BAPTA, and it was enhanced by increasing the extracellular Ca^2+^ concentration (Fig. 3c; Supplementary Fig. 3a). Increasing KCl concentration in the extracellular solution also promoted GlyR endocytosis and this KCl-induced effect was largely blocked by removing calcium from the extracellular solution, suggesting that calcium influx via VGCCs opened in response to membrane depolarization also triggers GlyR endocytosis (Fig. 3b). However, specific Ca^2+^ signatures resulting from KAR activation must also be important for the kainate-induced effects because neither NMDA nor AMPA induced a similar decrease in surface GlyR levels and the kainate-induced GlyR endocytosis was mediated by KARs but not AMPARs. This is not unusual as Ca^2+^ has pleiotropic effects on neurons that may lead to dramatically different downstream signalling events depending on the sources of the Ca^2+^ rise and activation of different Ca^2+^-dependent enzymes^58^,^59^. In this context, the spatiotemporal Ca^2+^ signal required to induce GlyR internalization in spinal cord neurons is generated by activation of KARs, but not NMDARs or AMPARs. This signal may be mimicked partially by KCl-induced VGCC activation, which lacks the spatiotemporal control as compared with receptor-mediated stimulation. Since kainate-induced GlyR endocytosis was only marginally attenuated by blocking L-type VGCCs with nifedipine (Supplementary Fig. 3b), the regulation of surface GlyRs by KAR activation is more likely mediated by Ca^2+^ influx through the KARs rather than L-type VGCCs, which also opens in response to KAR-elicited membrane depolarization.

Although KARs are also phosphorylated and functionally regulated by PKC^5^,^6^,^21^,^35^,^60^, including a PKC-dependent trafficking of GluK2-containing KARs to early endosomes and subsequent sorting into either recycling or degradation pathways^5^, the regulations of KARs and GlyRs by PKC may not share a common pathway. First, it is unlikely that KARs and GlyRs form physical signalling complexes that co-internalize on kainate stimulation in a similar manner as that described for NMDAR/dopamine receptor complexes^61^,^62^,^63^ or G protein-coupled receptor/calcium channel complexes^64^. We were unable to detect an obvious colocalization between the two receptor types either with or without the kainate treatment (Supplementary Fig. 5a) or co-immunoprecipitation of GlyRs and KARs from spinal cord lysates (Supplementary Fig. 5b). Second, the *SENP1*^−/−^ neurons had normal expression levels of GluK2 and the normal function of KARs (Supplementary Fig. 4b–d). The crosstalk between KARs and GlyRs requires not only intracellular Ca^2+^ elevations and PKC activation, but also SENP1-dependent deSUMOylation of the kinase. We detected SUMOylation of PKC in both heterologous system and cultured neurons and the SUMOylation levels of PKC in the neurons were decreased by kainate treatment (Fig. 6). Intriguingly, whereas the wild-type SENP1 abolished SUMOylation of PKC, the catalytically inactive SENP1 mutant was unable to remove SUMO from PKC, demonstrating that PKC deSUMOylation is one of the functions exerted by SENP1. To further establish the specificity of SUMO-PKC regulation on GlyR endocytosis, we showed that the diminished effect of kainate on GlyR internalization in *SENP1*^−/−^ neurons was rescued by overexpressing wild-type SENP1 (Supplementary Fig. 4f). In addition, overexpressing non-SUMOylatable PKCα mutant, K465R, in cultured neurons reduced cell surface GlyR levels under non-stimulated conditions and abolished the effect of kainate treatment on GlyR endocytosis. These suggest that the mutant mimicked the SENP1-mediated deSUMOylated state, normally associated with kainate stimulation (Fig. 7d; Supplementary Fig. 7).

It was recently shown that inhibiting network activity by TTX reduced SENP1 levels in cultured hippocampal neurons, leading to an increase in protein SUMOylation^39^ and neuronal depolarization elicited by high KCl concentrations caused a differential redistribution of SUMOylation/deSUMOylation enzymes in and out of synapses^26^, indicating that synaptic SUMOylation levels are dynamically regulated by neuronal activity. Therefore, it is predictable that the SUMOylation status of PKC will change in consequence to reduced SENP1 levels and/or redistribution of SENP1. Here we show that KARs induce GlyR endocytosis via a mechanism that involves calcium influx and SENP1-mediated deSUMOylation of PKC. Intriguingly, this effect of KARs was abolished by the treatment with TTX (Fig. 3a), which does not prevent calcium influx through KARs. It is possible that TTX altered the SUMOylation status of PKC via downregulation of SENP1 (ref. 39), just like in the case of SENP1 knockout, or it impaired the activity-dependent redistribution of SENP1 in the spinal cord neurons, which could be important for PKC deSUMOylation and consequent induction of GlyR endocytosis. Conversely, the induction of GlyR endocytosis by KCl could also be related to the redistribution of SUMOylation/deSUMOylation enzymes in and out of synapses evoked by this treatment, in addition to its effect on activation of VGCCs. Our study, however, does not rule out the possibility that SUMOylation of other proteins, as well as the involvement of other unidentified factors released or activated following kainate/KCl stimulation, in the kainate- (or KCl-) induced GlyR endocytosis. In addition, the contribution of presynaptic KARs remains to be ruled out and further studies are required to elucidate the detailed mechanism on activity-dependent regulation of GlyR endocytosis.

**Figure 9:**
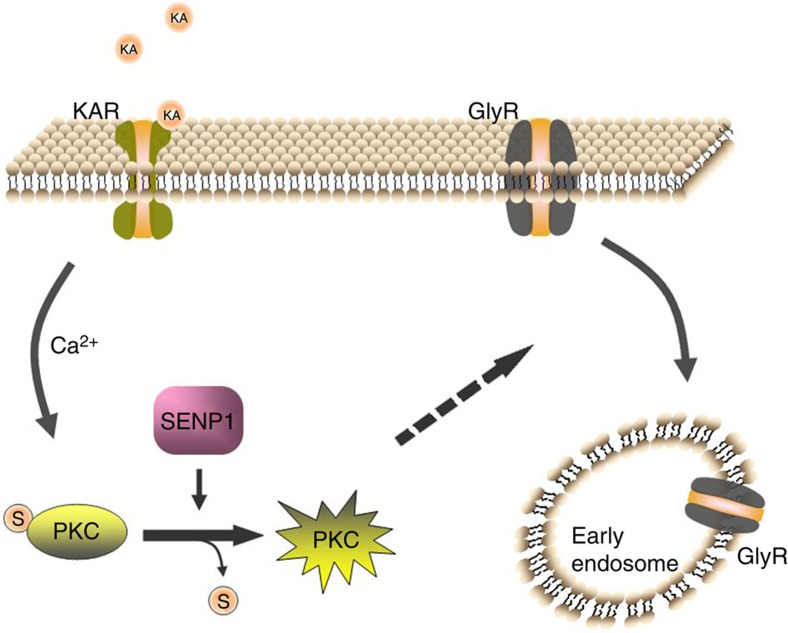
A model of crosstalk between KARs and GlyRs mediated by deSUMOylation of PKC. Ca^2+^ entry through activated KAR triggers activation of PKC depending on its SUMOylation status. DeSUMOylation of PKC by SENP1 allows it to be activated by Ca^2+^, leading to consequent endocytosis of GlyRs.

On the basis of the findings reported here, we propose a model for SENP1 in the regulation of kainate-induced GlyR endocytosis through a pathway that depends on SENP1-mediated deSUMOylation of PKC (Fig. 9). The activation of classical PKCs in response to Ca^2+^ influx through activated KARs is dependent on the SUMOylation status of the PKCs. Only when the PKCs are deSUMOylated through SENP1, can they be activated and phosphorylate GlyRs to trigger receptor endocytosis. In the absence of SENP1, PKC is unable to be deSUMOylated and thus unable to be activated by the KAR pathway to induce GlyR internalization.

The inhibitory GlyRs are ligand-gated chloride channels that mediate fast synaptic inhibition in spinal cord and brainstem^7^,^8^ at glycinergic^10^,^11^,^12^ or mixed glycinergic/GABAergic postsynaptic sites^14^,^15^. Conversely, KARs regulate synaptic transmission and neuronal excitability in the mammalian brain by acting at pre-, post- and extrasynaptic sites, depending on their specific localization and density at these three sites^5^,^6^. In this context, the downregulation of GlyRs as a result of KAR activation is expected to amplify the excitatory effects mediated by glutamate receptor activation. This anti-homeostatic modulation between KARs and GlyRs may provide a physiological means for maintaining proper neuronal excitability through alteration of excitatory-inhibitory balance. Our experiments thus far provide the first evidence that PKC deSUMOylation plays a role in this unique modulatory function on neuronal excitability.

## Methods

### Primary spinal cord neuron cultures

All animal procedures were carried out in accordance with the guidelines for the Care and Use of Laboratory Animals of Shanghai Jiao Tong University School of Medicine and approved by the Institutional Animal Care and Use Committee. The culture procedure for dissociated neurons was as described previously^65^ with the following modifications. The generation and screening of *SENP1*^−/−^ foetuses was described previously^66^ and *SENP1*^−/−^ mice were maintained by crossing heterozygous males and females. Briefly, embryos were removed from anaesthetized embryonic day (E) 13.5 Sprague-Dawley rats or E13.5 SENP1 heterozygous mice. Spinal cords were dissected in cold HBSS medium supplemented with 10 mM HEPES and 1% penicillin/streptomycin (Invitrogen), and the meninges were carefully removed. The spinal cords were chopped into small pieces and then trypsinized for 15 min and mechanically dissociated at 37 °C. A stop buffer was composed of DMEM supplemented with 10% heat-inactivated fetal calf serum (Invitrogen) and added to cells when trypsin digestion was completed. After trituration, cells were spun down at 800 r.p.m. for 10 min, resuspended in 10 ml of neuronal feed (neurobasal medium supplemented with B27 and 2 mM L-glutamine (Invitrogen)) and then placed in a 60-mm culture dish for 10 min. Cell suspension was carefully collected for cell counting. Neurons were plated at a density of 500,000 per 35-mm dish or 150,000 onto 13-mm glass coverslips (VWR international) coated sequentially with 0.01% poly-ornithine (Sigma) and 10 μg ml^−1^ fibronectin (Invitrogen) and allowed to grow at 37 °C in a 5% CO_2_ incubator until use. Cultured spinal cord neurons were transfected with appropriate complementary DNAs after culturing for 6–8 days *in vitro* using the modified calcium phosphate transfection method and used for experiments at least 48 h post transfection.

### Antibody-feeding assay under non-permeabilized conditions

Antibody-feeding assay under non-permeabilized conditions were performed as described^67^,^68^ with modifications. Briefly, live spinal cord neurons 8–10 days *in vitro* were labelled for 45–60 min at 37 °C with an antibody directed against the extracellular region of GlyR (Abcam or SYSY) in neuronal feed and then washed with warm neuronal feed for three times. Pharmacological agents were added along with primary antibody (anti-GlyR) for 45–60 min in neuronal feed before kainate stimulation. After washing, neurons were treated with kainate at desired concentrations at 37 °C for 1 min or together with respective agents in feeding medium. Subsequently, neurons were fixed immediately for 15 min at room temperature in 4% paraformaldehyde/4% sucrose without permeabilization, and stained with goat anti-mouse or goat anti-rabbit Alexa Fluor 488-conjugated secondary antibodies (Invitrogen, 1:600 dilution) for 1 h at room temperature, to label surface receptors. Neurons were then permeabilized for 1 min in 100% methanol at −20 °C and stained with MAP2 antibody (SYSY, 1:800 dilution) and goat anti-guinea pig Alexa Fluor 633-conjugated secondary antibodies (Invitrogen, 1:600 dilution) for 1 h at room temperature, to visualize dendritic morphology. For Fig. 1B, antibody-feeding assay was carried out to the point of methanol permeabilization, and then the internalized GlyR neurons were stained with goat anti-mouse Alexa Fluor 546-conjugated secondary antibodies (Invitrogen, 1:600 dilution) for 1 h at room temperature to visualize pre-labelled internalized GlyRs before stained with MAP2 antibody to show dendritic morphology. Fluorescence images were acquired using a Zeiss LSM 510 oil immersion × 40 objective with sequential acquisition setting and quantified with Image J software. For fluorescence quantification, images were acquired using the same settings below saturation at a resolution of 1024 × 1024 pixels (12 bit). To count number of green puncta (GlyR puncta) and calculate green (GlyR) fluorescent intensities, MAP2 signal was used to generate mask and outline for dendritic structure in ImageJ, and set appropriate stroke size for dendritic outline to quantify surface-remaining GlyR. In addition, to exclude the influence of cell size, quantification of surface-remaining GlyR was measured as total fluorescent intensity/the area of MAP2 marked dendritic structure and normalized by untreated controls.

### Colocalization of internalized GlyRs with endosomal markers

The antibody-feeding assay was carried out as described above. Briefly, the primary antibody against the extracellular domain of GlyR was added into culturing medium for both control and kainate-stimulated groups to pre-label surface receptors on live neurons. Cells were then fixed with 4% paraformaldehyde/4% sucrose for 15 min at room temperature and permeabilized for 1 min in 100% methanol at −20 °C. Cells were incubated with the primary antibody against the early endosome marker EEA1 (Cell Signaling Technology, 1:100 dilution) overnight at 4 °C and then labelled with goat anti-mouse Alexa Fluor 488-conjugated secondary antibody (1:600 dilution) to visualize pre-labelled GlyRs (both internalized and surface-remaining) and goat anti-rabbit Alexa Fluor 546-conjugated secondary antibodies (1:600 dilution) to reveal early endosomal positive intracellular compartments.

### Surface biotinylation assay

Surface biotinylation was performed on cultured spinal cord neurons following established protocols^69^ with minor modification. Neurons were first stimulated with 200 μM kainate for 1 min at 37 °C. Cells were washed three times with the ice-cold PBS+/+ solution (PBS plus 1 mM MgCl_2_ and 2.5 mM CaCl_2_), followed by addition of 0.25 mg ml^−1^ Sulfo-NHS-LC-Biotin (Thermo Scientific) in the PBS+/+ solution at 4 °C for 30 min with gentle rocking. Unbound biotin group was quenched by a PBS+/+ solution that contained 0.1 M glycine. Total (T) proteins were extracted and incubated overnight at 4 °C with NeutrAvidin agarose beads (Thermo Scientific). The beads were washed three times with PBS+/+ and bound proteins were eluted with the boiling SDS sample buffer for western blot. Full images of western blots are shown in Supplementary Fig. 8.

### Ca^2+^ imaging and modulation of endocytosis by Ca^2+^

Neurons were rinsed before being loaded with Fluo-3 AM (2–5 μM, Invitrogen) in neuronal feed for 30–45 min at 37 °C, and then incubated for another 30 min after being rinsed in neuralbasal medium (Gibco) twice. Imaging was performed on a Zeiss LSM 510 confocal microscope with a × 40 oil objective (Zeiss Plan-Neofluar, numerical aperture 1.3). Cells were excited with 488 nm laser, and the intensity of the fluorescence emission between 505 and 550 nm was measured at 2 s intervals. The changes in Ca^2+^ signal were normalized to the baseline value before kainate treatment. Kainate was applied via a micropipette located at the centre of the coverslip by diluting a high-concentration stock (4 mM) into the extracellular solution to achieve a final concentration of 200 μM.

To test the effect of extracellular Ca^2+^ on kainate-induced GlyR internalization, neurons were pre-incubated either with neuronal feed containing 6 mM CaCl_2_ (plus 2 mM Ca^2+^ included in the commercial neurobasal medium, hence the total extracellular Ca^2+^ concentration was 8 mM), or neuronal feed containing cell-permeable Ca^2+^ chelator BAPTA-AM (50 μM), neuronal feed containing 2 mM EGTA to chelate extracellular calcium or an extracellular medium containing 2 mM EGTA with nominally free Ca^2+^. The medium composition was maintained during incubation with the anti-GlyR primary antibody and kainate stimulation for the antibody-feeding assay under non-permeabilized conditions.

### PKC kinase activity assay

PKC activity was measured using the PKC kinase activity kit according to the manufacturer’s instructions (ADI-EKS-420A, Enzo Life Sciences), which is based on a solid-phase enzyme-linked immunosorbent assay that utilizes a specific synthetic peptide as a substrate for subtypes of PKCs and a polyclonal antibody that recognizes the phosphorylated form of the substrate. The absorbance was measured at a wavelength of 450 nm and PKC activity was expressed as a relative activity.

### Spinal cord slice preparation and patch-clamp recordings

The lumbar segments of spinal cords were dissected from male Sprague-Dawley rats (2–3-week old) as described previously^70^ with minor modifications. A portion of the lumbar spinal cord (L4-L5) was removed from young male Sprague-Dawley rats (2–3-week old) under urethane anaesthesia and kept in pre-oxygenated ice-cold Kreb’s solution that contained (in mM): NaCl 118, KCl 2.5, CaCl_2_ 2.5, MgCl_2_ 1.3, NaH_2_PO_4_ 1.2, NaHCO_3_ 26 and glucose 11 (pH 7.4). Each spinal segment was placed in a shallow groove formed in an agar block and glued to the bottom of the microslicer stage. Transverse slices (350–400 μm) were cut with a Leica VT1200 S (Leica Microsystems) vibrating microslicer. The slices were perfused with the Kreb’s solution that was saturated with 95% O_2_ and 5% CO_2_ at 35±1 °C for at least 1 h before electrophysiological experiments.

Slices were moved to a glass-bottomed recording chamber mounted on the stage of an upright microscope (Olympus) equipped with infrared DIC (differential interference contrast), and held with a grid of nylon threads. The whole-cell patch-clamp recordings were made from spinal dorsal horn neurons in voltage-clamp mode^70^. Patch pipettes were fabricated from thin-walled, borosilicate, glass-capillary tubing (1.5 mm o.d. (outside diameter), World Precision Instruments) using a P-97 pipette puller (Sutter Instruments). After establishing the whole-cell configuration, neurons were held at −70 mV for recording sIPSCs. The resistance of a typical patch pipette was 3–5 MΩ when filled with the internal solution that contained (in mM): KCl 130, NaCl 5, CaCl_2_ 1, MgCl_2_ 2, EGTA 11, HEPES 10, Mg-ATP 2, Li-GTP 1, pH 7.2 adjusted by Tris-base. The external recording solution was the Kreb’s solution gassed with 95% O_2_ and 5% CO_2_. Membrane currents were amplified with an Axopatch 200A amplifier (Molecular Devices) in voltage-clamp mode. Signals were filtered at 2 kHz and digitized at 5 kHz via Digidata 1322A. Data were stored on a personal computer using pCLAMP 10 software and analysed with Mini Analysis (Synaptosoft) and ORIGIN for Windows (Microcal Software).

All drugs were obtained from Sigma. Receptor agonists or antagonists were diluted with the Kreb’s solution to final desired concentrations and applied via the ‘Y-tube’ method as described elsewhere^65^. The tip of the drug tube was positioned between 50 and 100 μm away from the patched neurons. This system allows a complete exchange of external solution surrounding a neuron within 20 ms. Throughout all experimental procedures, the bath was continuously perfused with the standard external solution.

### Plasmids and antibodies

Flag-SUMO1, RGS-SENP1 and RGS-SENP1m were gifts from Jinke Cheng (Shanghai Jiao Tong University School of Medicine, Shanghai, China). HA-PKCα was purchased from Addgene. The following primary antibodies and dilutions were used: Mouse Anti-RGS (34610, 0.1 μg ml^−1^ dilution) was from Qiagen. Mouse Anti-Flag (M2, F3165, 1:1,000), Mouse Anti-PKC (P5704, 1:500), Mouse Anti-HA (H9658, 1:2,000) and Anti-Flag M2 affinity gel were from Sigma. Rabbit Anti-SUMO1 (4940, 1:1,000), Rabbit Anti-MARCKS (1:1,000), Rabbit Anti-p-MARCKS (1:1,000) and Rabbit Anti-PKCα (1:1,000) were purchased from Cell Signaling. Rat Anti-HA (1:2,000) was purchased from Roche. Anti-HA agarose was from Pierce Biotechnology. Anti-GlyR was from Abcam (rabbit, 1:300) or Synaptic Systems (mouse, 1:300). Anti-PKC antibodies (sc-17804, A9, mouse, 1:500; sc-10800, H-300, rabbit 1:500) were from Santa Cruz Biotechnology.

### Co-immunoprecipitation

Homogenates of P14 (postnatal day 14) rat spinal cords were generated in the presence of proteinase inhibitor cocktail Complete (Roche) in an ice-cold buffer (50 mM Tris–HCl, pH 7.4, 150 mM NaCl and 1% Triton X-100) and subjected to end-to-toe rotation at 4 °C for 30 min. Detergent extracts were centrifuged at 13,000*g* for 20 min at 4 °C, the concentration of the solubilized protein was determined with a BCA assay kit (Thermo Scientific) and analysed by an Eppendorf Master Photometer. Solubilized spinal cord homogenates (1 mg proteins) were incubated with 5 μg of monoclonal antibodies against GlyRs (Synaptic Systems) or control mouse IgG (Sigma) in 1,000 μl of Tris-buffered saline with 0.1% Triton X-100 and protease inhibitors, and placed on a microtube rotator at 4 °C for 3 h. Protein A-Sepharose CL-4B resin (2.5 mg; Amersham Biosciences) was added to each sample, and the incubation continued for an additional 3 h, followed by three washes with Tris-buffered saline/0.1% Triton X-100. The immobilized protein complexes were processed for SDS–PAGE (Invitrogen) and immunoblotting using enhanced chemiluminescence procedures (Amersham Biosciences). Anti-GlyR (Abcam) and anti-GluR6 (AnaSpec) antibodies were used in the immunodetection. For *in vivo* SUMOylation assay, CHO-K1 cells were transfected with indicated plasmids. At 24 h after transfection, cells were lysed with immunoprecipitation buffer (10 mM phosphate buffer, 10 mM Tris, 150 mM NaCl, 1% Triton X-100 and 20 mM NEM (N-Ethylmaleimide) (pH 7.5)) containing a protease inhibitor cocktail, and immunoprecipitated with the indicated antibodies. Immunoprecipitated proteins were resolved by SDS/PAGE and analysed by immunoblotting. Full images of western blots are shown in Supplementary Fig. 8.

### Statistical analysis

Statistical analyses of antibody-feeding assay under non-permeabilized conditions were performed using ImageJ software. Data are expressed as means±s.e.m., with statistical significance assessed by Student’s *t*-test for two-group comparison or one-way analysis of variance tests for multiple comparisons. The value of *P*<0.05 was considered to have statistically significant difference.

## Author contributions

Y.L. supervised the project and designed the experiments. H.S. and L.L. conducted the majority of imaging experiments. Y.J. performed and analysed the electrophysiological experiments. H.S., Y.W., W.-Z.Z., C.H. and Y.Z. performed the biochemical experiments. M.X.Z., G.W.Z., T.Z., T.-L.X. and J.C. discussed the results. Y.L. wrote the manuscript.

## Additional information

**How to cite this article:** Sun, H. *et al*. Kainate receptor activation induces glycine receptor endocytosis through PKC deSUMOylation. *Nat. Commun.* 5:4980 doi: 10.1038/ncomms5980 (2014).

## Supplementary Material

Supplementary Figures Supplementary Figures 1-8
