# Supplementary material for: Kainate receptor activation induces glycine receptor endocytosis through PKC deSUMOylation
Source: Nat Commun. 2014 Sep 19;5:4980. doi: 10.1038/ncomms5980 (PMC4199113; doi:10.1038/ncomms5980)
Supplement: Supplementary Figures — 1-8 [file ncomms5980-s1.pdf]

Supplementary Figures

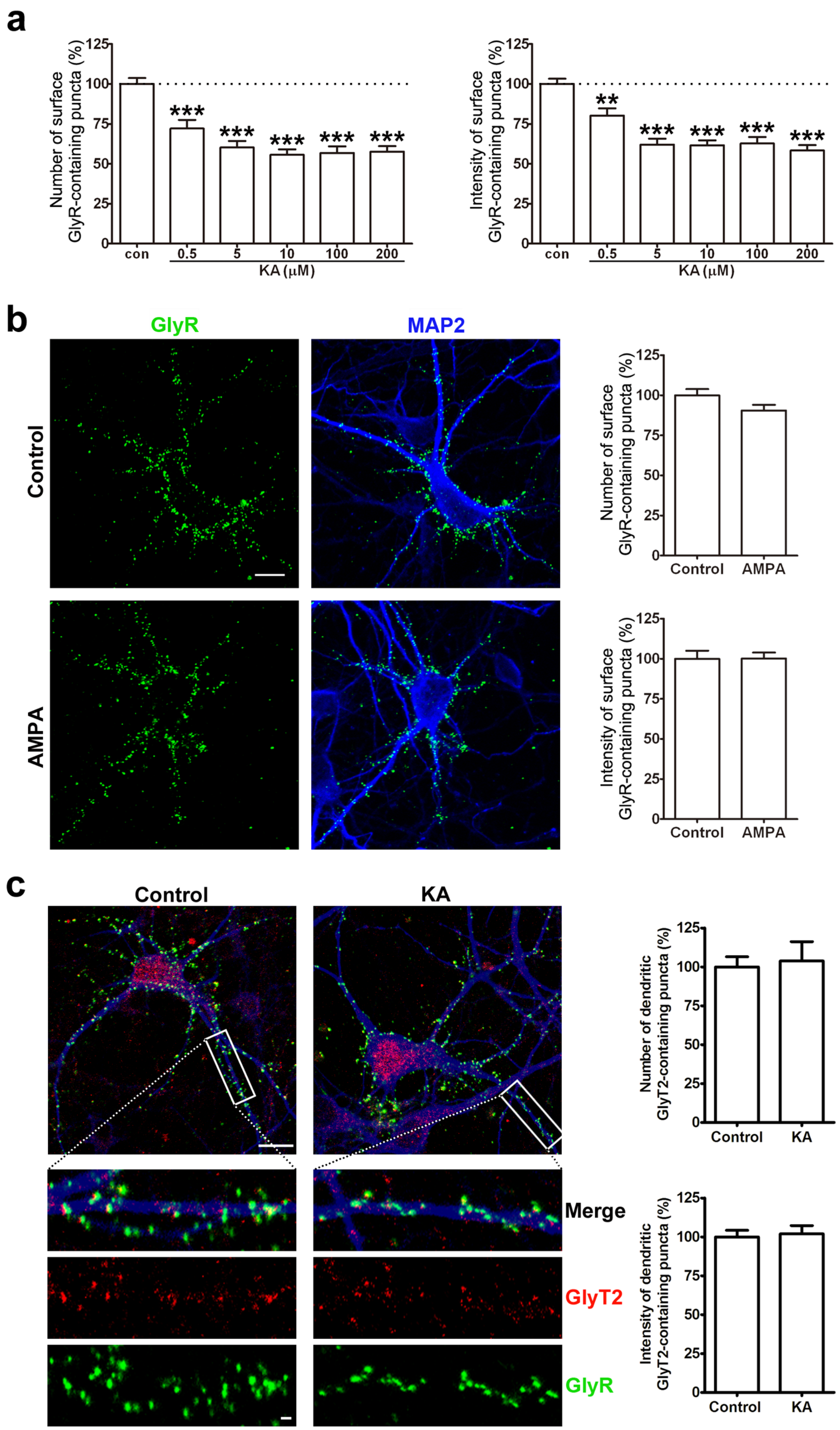

Supplementary Figure 1. Effects of different concentrations of kainate and AMPA on GlyR endocytosis. (**a**) Histogram illustrating dose dependence decreases in the puncta number (*left*) and intensity (*right*) of surface GlyRs by kainate (KA) stimulation. Data are means ± SEM from at least three experiments; the total numbers of neurons analyzed (n) ranged from 22 to 84 cells per condition. (**b**) Antibody feeding assay under non-permeabilized conditions labeled cell surface GlyRs (*green*) in cultured rat spinal cord neurons untreated (Control) or briefly treated for 1 min with 100 μM AMPA. Neurons were then permeabilized and labeled with MAP2 (*blue*) to show cell body and processes. Puncta number (*upper*) and integrated fluorescence intensity (*lower*) of surface GlyRs from all images analyzed by ImageJ and values normalized to untreated controls. Data are means ± SEM from five experiments; the total numbers of neurons analyzed (n) ranged from 47 to 54 cells per condition. Bar, 10 μm. **, $P < 0.01$, ***, $P < 0.001$ compared to control, by one-way ANOVA with pairwise comparison by Tukey's post hoc test. (**c**) Double immunofluorescence labeling of presynaptic glycine transporter 2 (GlyT2, *red*) and GlyR (*green*) of neurons untreated (Control) or treated with kainate (KA) for 1 min. Magnified views of boxed areas are shown in the lower panels. Bars, 10 μm (original, *upper*) and 1 μm (magnified, *lower*). Data are means ± SEM from three experiments; the total numbers of neurons analyzed (n) 11 cells per condition.

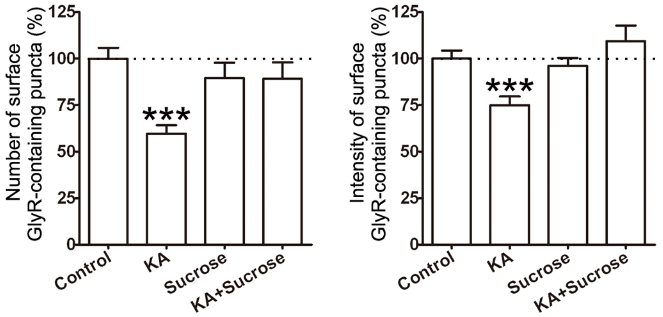

Supplementary Figure 2. Effect of Hypertonic sucrose on GlyR endocytosis. GlyR endocytosis induced by kainate (KA) in rat spinal cord neurons was abolished by treatment with 0.45 M sucrose. Quantification data are shown as means ± SEM from at least two experiments; the total numbers of neurons analyzed (n) ranged from 15 to 33 cells per condition. ***, $P < 0.001$ compared to control, by one-way ANOVA with pairwise comparison by Tukey's post hoc test.

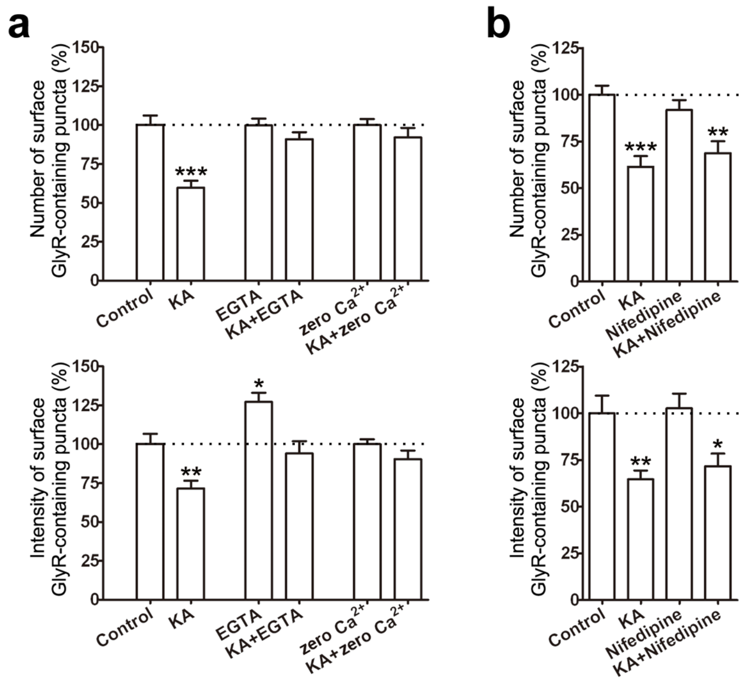

Supplementary Figure 3. Kainate-induced GlyR internalization is calcium-dependent. (**a**) Kainate (KA)-induced GlyR internalization was inhibited by either chelation of extracellular calcium with EGTA or removing calcium from the extracellular medium (nominally calcium free). Quantification data show means ± SEM of surface GlyR cluster numbers and intensities from at least three experiments; the total numbers of neurons analyzed (n) ranged from 24 to 42 cells per condition. (**b**) Kainate (KA)-induced GlyR internalization was not significantly attenuated by nifedipine. Quantification data show means ± SEM of surface GlyR cluster numbers and intensities from at least two experiments; the total numbers of neurons analyzed (n) ranged from 9 to 12 cells per condition. *, $P < 0.05$, **, $P < 0.01$, ***, $P < 0.001$ compared to control, by one-way ANOVA with pairwise comparison by Tukey's post hoc test.

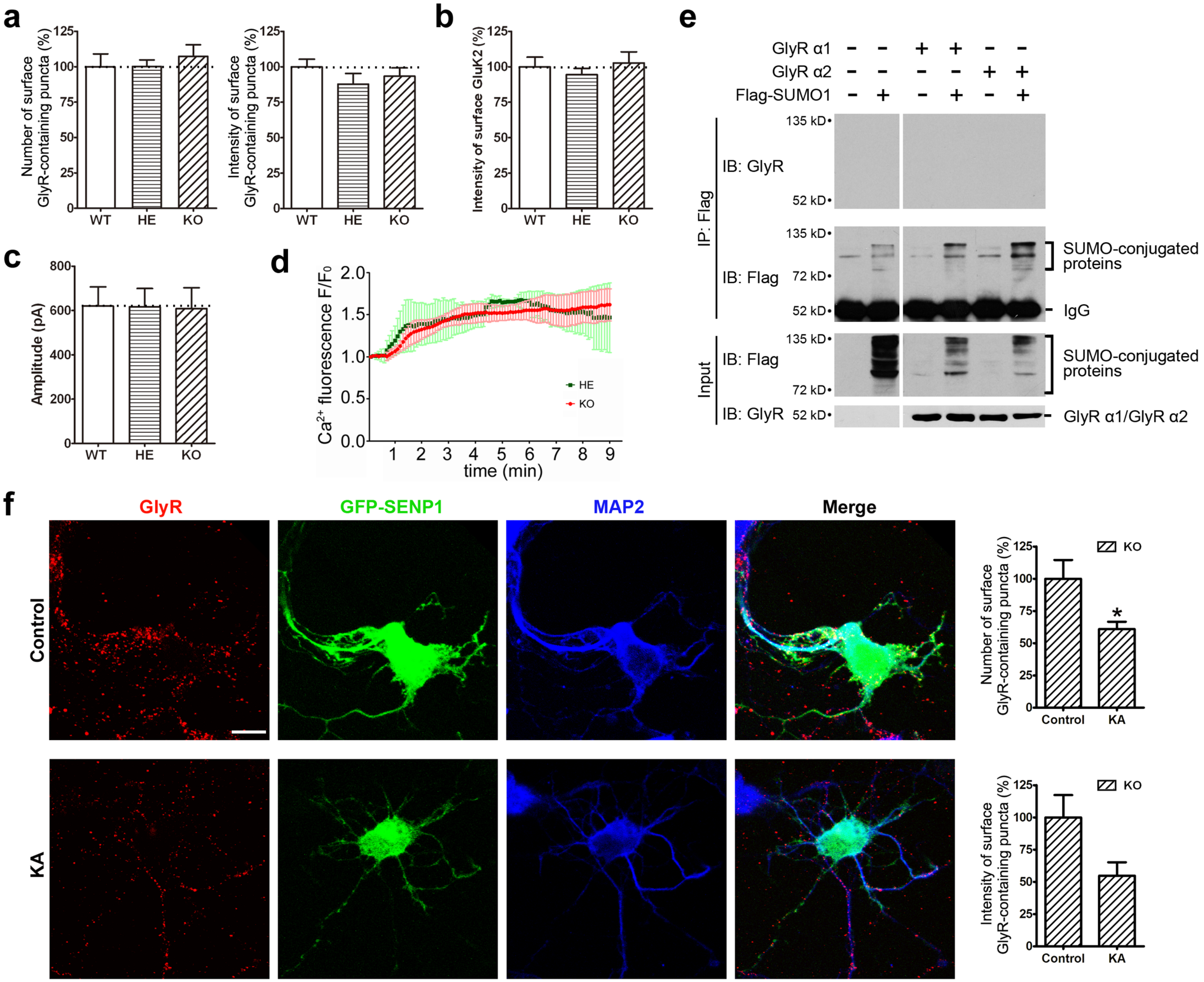
a
Number of surface GlyR-containing puncta (%)
Intensity of surface GlyR-containing puncta (%)
WT
HE
KO
b
Intensity of surface GluK2 (%)
c
Amplitude (pA)
d
Ca2+ fluorescence F/F0
time (min)
HE
KO
e
GlyR α1
GlyR α2
Flag-SUMO1
IP: Flag
IB: GlyR
IB: Flag
Input
IB: Flag
IB: GlyR
135 kD
72 kD
52 kD
SUMO-conjugated proteins
IgG
GlyR α1/GlyR α2
f
GlyR
GFP-SENP1
MAP2
Merge
Control
KA
Number of surface GlyR-containing puncta (%)
Intensity of surface GlyR-containing puncta (%)
KO
Control
KA
*

Supplementary Figure 4. *SENP1$^{-/-}$* neurons have normal expression levels of GlyRs and GluK2 and the normal function of KARs and GlyRs are not modified by SUMO1 in vivo. (**a**) Histograms depict the puncta number and intensity of surface GlyRs as quantified by the antibody feeding assay under non-permeabilized conditions in *SENP1$^{+/+}$* (WT), *SENP1$^{+/-}$* (HE) and *SENP1$^{-/-}$* (KO) neurons as indicated. Data are normalized to WT and shown as means ± SEM from three experiments; the total numbers of neurons analyzed (n) ranged from 20 to 23 cells per condition. (**b**) Histograms depict the intensity of GluK2-containing KARs in *SENP1$^{+/+}$* (WT), *SENP1$^{+/-}$* (HE) and *SENP1$^{-/-}$* (KO) neurons as indicated. Data are normalized to WT and shown as means ± SEM from three experiments; the total numbers of neurons analyzed (n) ranged from 18 to 22 cells per condition. (**c**) Bar graph showing means ± SEM of the amplitudes of kainate-activated currents (I_{KA}) in *SENP1$^{+/+}$* (WT), *SENP1$^{+/-}$* (HE) and *SENP1$^{-/-}$* (KO) neurons. Data are normalized to WT and from three experiments; the total numbers of neurons analyzed (n) ranged from 10 to 14 cells per condition. (**d**) Spinal cord neurons from *SENP1$^{+/-}$* (HE) and *SENP1$^{-/-}$* (KO) embryos were loaded with Fluo-3 AM at 8-10 DIV and then calcium imaging were taken to detect the KAR-mediated Ca^{2+} transients elicited by kainate. Non-KAR components of Ca^{2+} influx was blocked by including CNQX (20 μM) and APV (50 μM) in the extracellular solution. Colored bars show the F/F_0 scale (where F is instantaneous fluorescence at a given time point and F_0 is baseline fluorescence). Data are from three experiments; the total numbers of neurons analyzed (n) ranged from 3 to 5 neurons for each genotype. (**e**) GlyRα1, GlyRα2 and Flag-tagged SUMO1 were expressed in HEK293T cells as indicated. Cell lysates were immunoprecipitated using anti-Flag and analyzed by immunoblotting using GlyR and Flag antibodies as indicated. No GlyR was detected in the Flag immunoprecipitants (for SUMOylated proteins). (**f**) Surface GlyRs assessed by the antibody feeding assay under non-permeabilized conditions in neurons prepared from *SENP1$^{-/-}$* (KO) mice by overexpressing GFP-SENP1. Quantification data are means ± SEM from two experiments; the total numbers of neurons analyzed (n) 5 cells per condition. Bar, 10 μm. *, $P < 0.05$, compared to control by Student's t test P=0.0317.

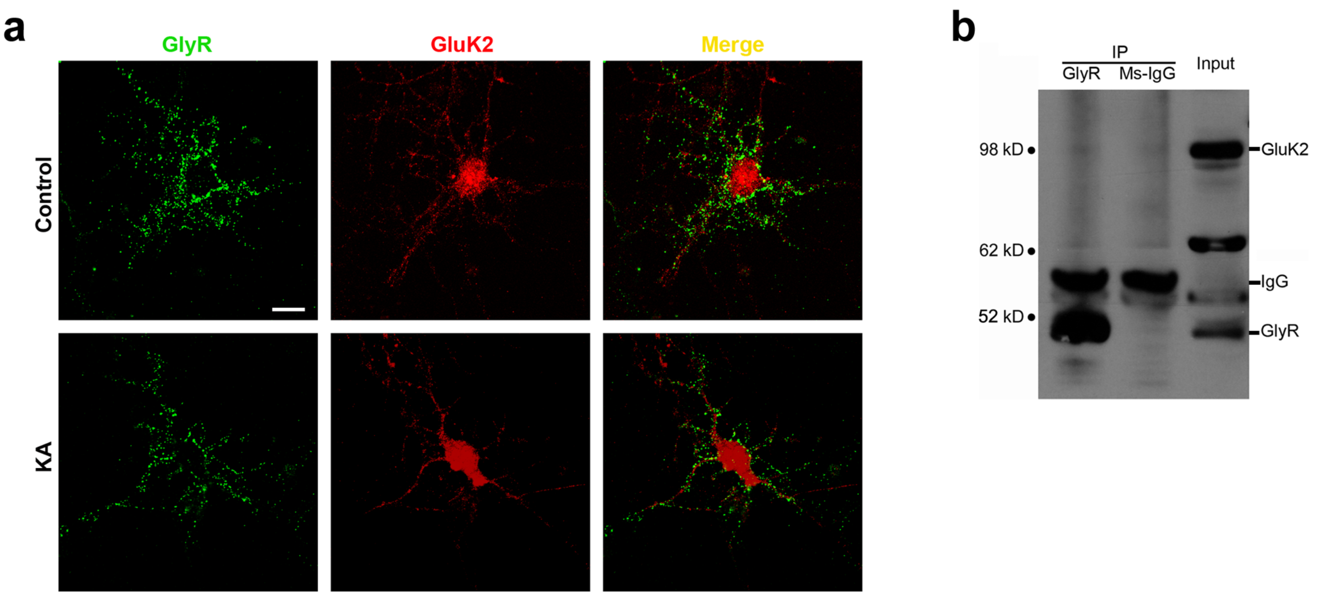

Supplementary Figure 5. GlyR and GluK2 do not colocalize or form protein complexes in rat spinal cord neurons. (**a**) GlyR and GluK2 do not co-localize in spinal cord neurons. Double immunostaining of GlyR (*green*) and GluK2 (*red*) revealed that most of GlyR clusters on the surface do not co-localize with GluK2 as assessed after permeablization of the cells. (**b**) Lack of a protein complex composed of GluK2 and GlyR in spinal cord. GluK2 (102 kD and also 66 kD isoform) was not co-immuoprecipitated with GlyRs from rat spinal cord homogenate. The 48 kD GlyRs were successfully precipitated by the anti-GlyR antibody.

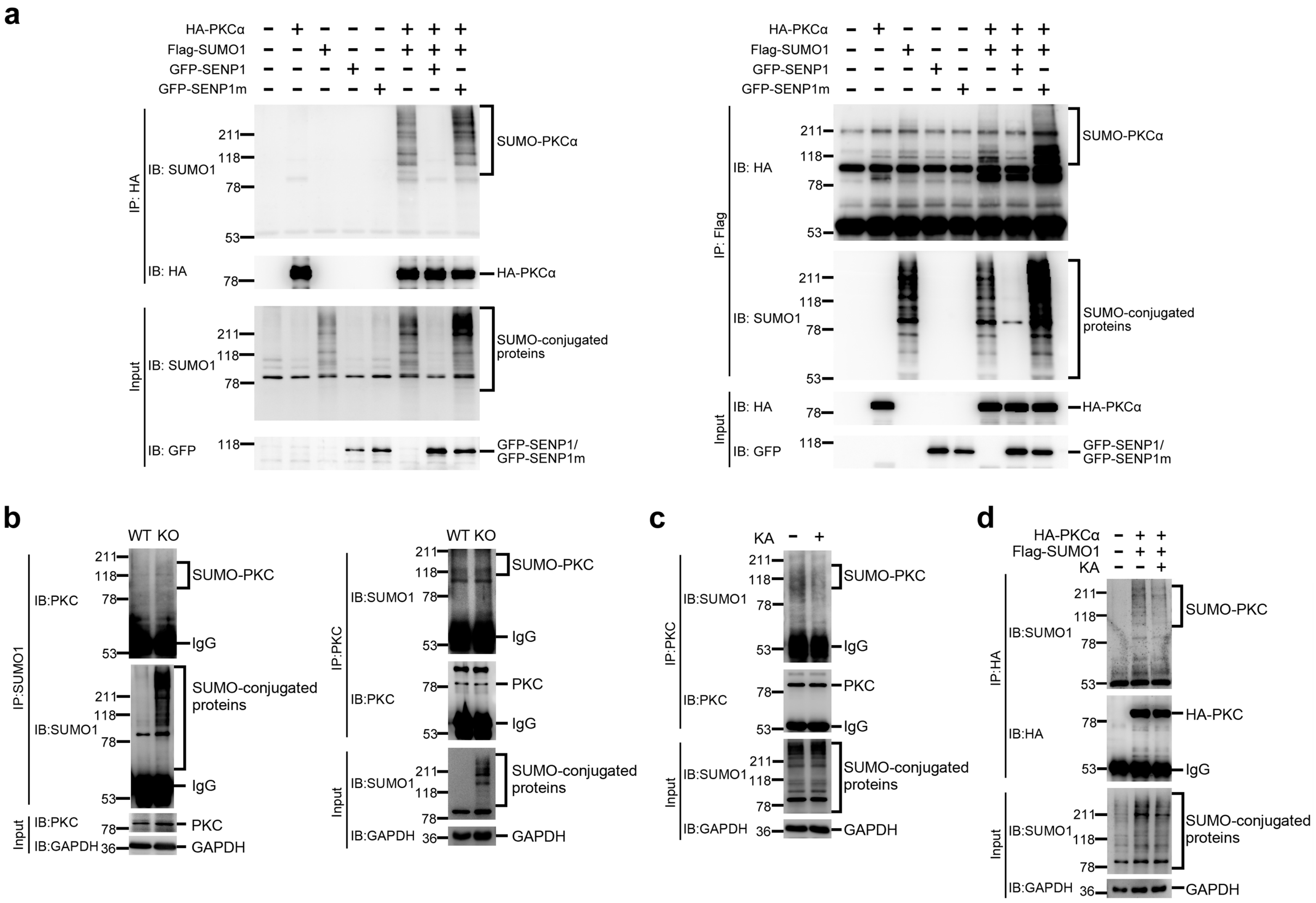
a
HA-PKCα
Flag-SUMO1
GFP-SENP1
GFP-SENP1m
IP: HA
IB: SUMO1
IB: HA
Input
IB: SUMO1
IB: GFP
SUMO-PKCα
HA-PKCα
SUMO-conjugated proteins
GFP-SENP1/ GFP-SENP1m
IP: Flag
IB: HA
IB: SUMO1
b
WT KO
IP:SUMO1
IB:PKC
IB:SUMO1
IB:GAPDH
SUMO-PKC
IgG
PKC
GAPDH
IP:PKC
c
KA
d
IP:HA
HA-PKC

Supplementary Figure 6. PKC SUMOylation under denaturing conditions. (**a-c**) PKC SUMOylation under denaturing conditions. (**a**) CHO-K1 cells were transfected with HA-tagged PKC, Flag-tagged SUMO1, GFP-tagged SENP1 or GFP-tagged SENP1m (inactive mutant) as indicated. Cells were lysed in a buffer containing 1% SDS and lysates immunoprecipitated (IP) with either anti-HA (*left*) or anti-Flag antibodies (*right*). Immunoblotting (IB) was performed using either anti-SUMO1 or anti-HA antibodies as indicated. (**b**) Similar to (**a**) except tissue homogenates from *SENP1*$^{+/+}$ and *SENP1*$^{-/-}$ embryos were lysed in the 1% SDS buffer and immunoprecipitation carried out by either anti-PKC (*left* panel) or anti-SUMO1 antibodies (*right* panel). Immunoblotting was performed using anti-SUMO1 or anti-PKC antibodies as indicated. (**c**) Similar to (**a**) except cultured spinal cord neurons from wild type neurons untreated (-) or treated (+) with kainate (KA, 200 μM, 1 min) were lysed in the 1% SDS buffer and immunoprecipitation carried out by anti-PKC antibodies. Immunoblotting was performed using anti-SUMO1 or anti-PKC antibodies as indicated. Data are representative of at least three independent experiments. (**d**) Neurons transfected with HA-PKCα and Flag-SUMO1 were untreated (-) or treated (+) with kainate (KA, 200 μM, 1 min) and immunoprecipitated using anti-HA antibodies, followed by immunoblotting using anti-SUMO1 antibodies as indicated. Data are representative of at least three independent experiments.

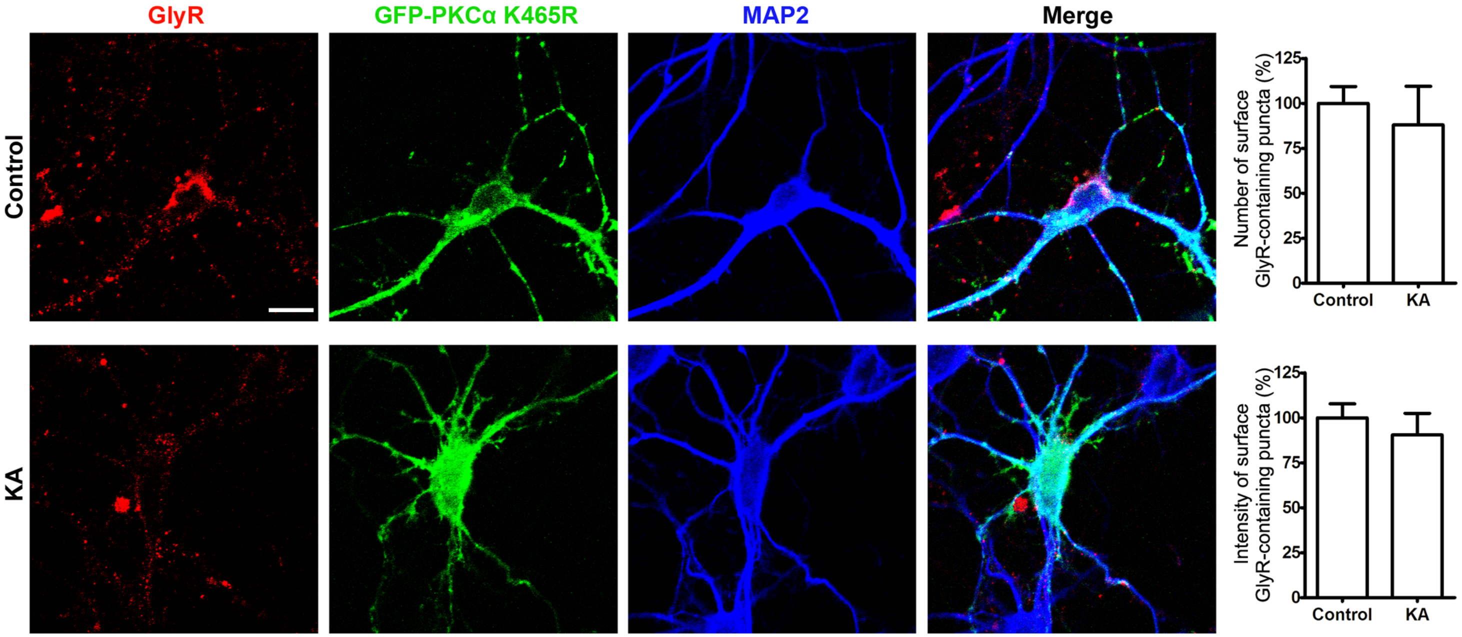

Supplementary Figure 7. Overexpression of PKCα K465R mutant attenuated the effect of kainate-induced GlyR endocytosis. Surface GlyRs assessed by the antibody feeding assay under non-permeabilized conditions from control and kainate (KA)-treated cultured spinal cord neurons overexpressed GFP-PKCα K465R. Quantification data are means ± SEM from three experiments; the total numbers of neurons analyzed (n) ranged from 7 to 8 cells per condition. Bar, 10 μm.

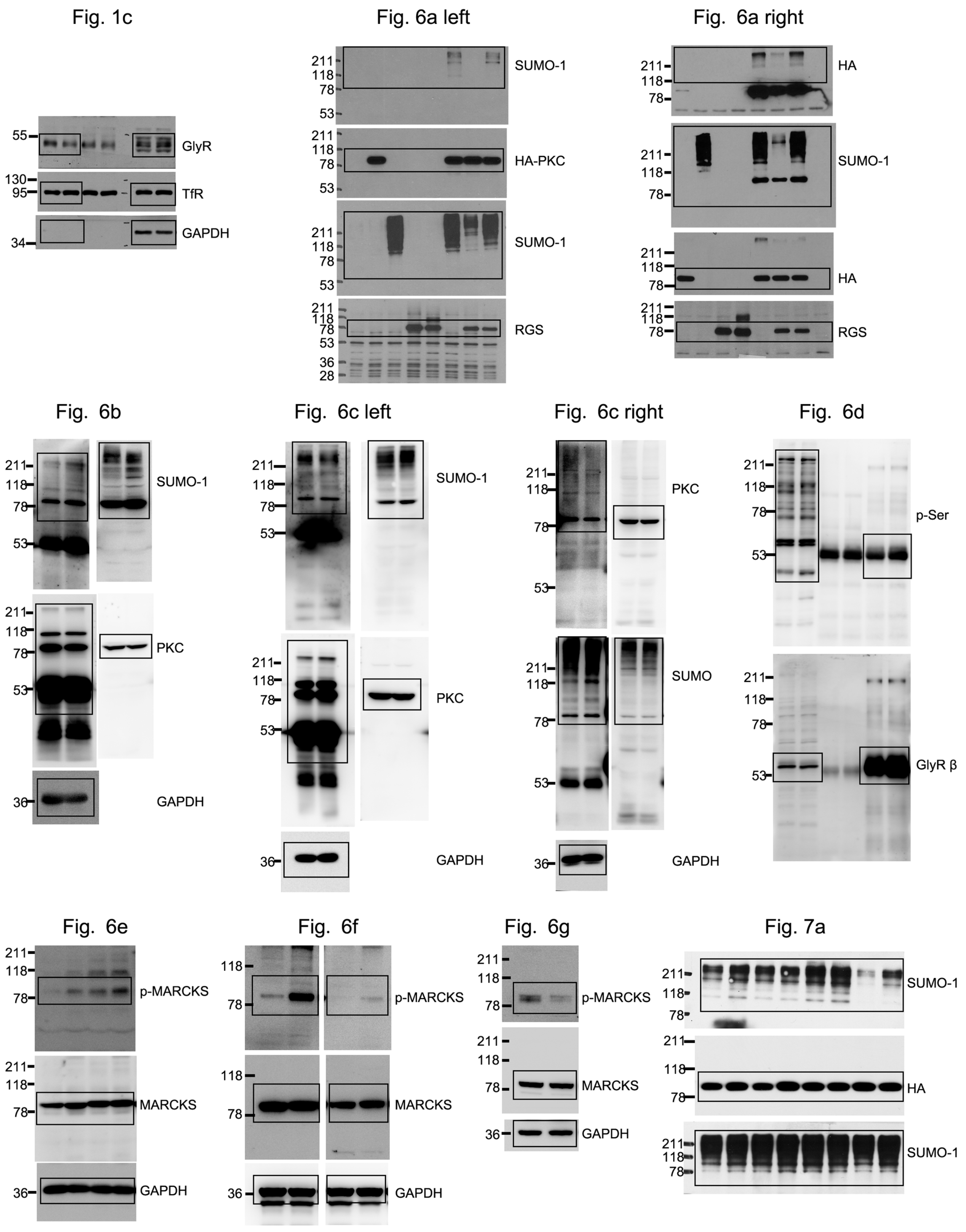

Supplementary Figure 8. Full images of western blots.

Supplementary Fig. 4e

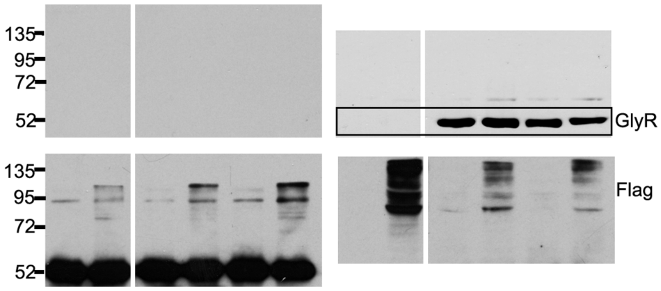

Supplementary Fig. 5b

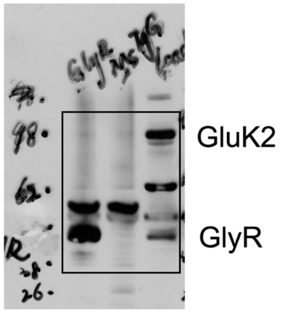

Supplementary Fig. 6a left

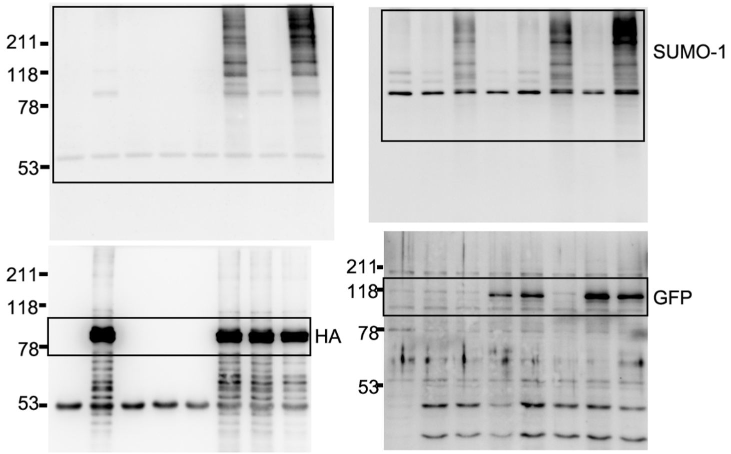

Supplementary Fig. 6b

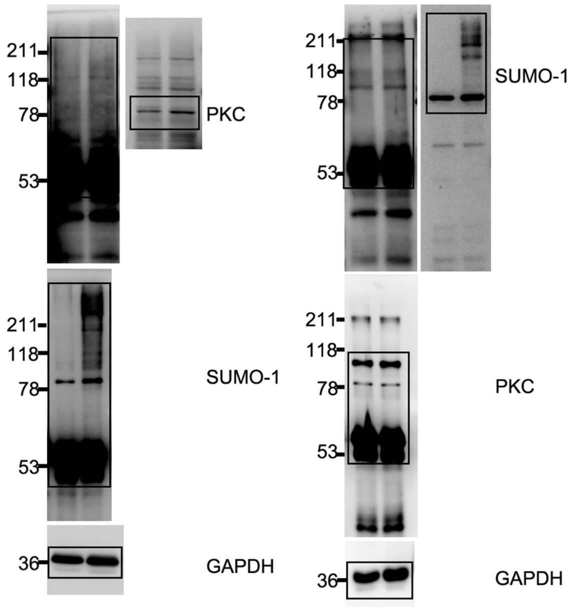

Supplementary Fig. 6a right

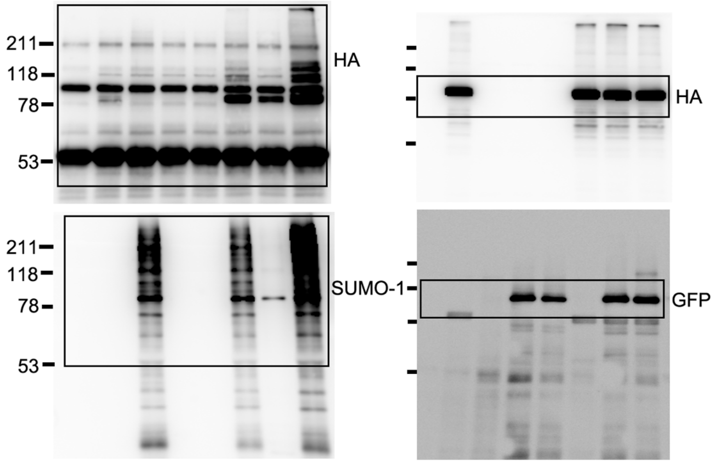

Supplementary Fig. 6c

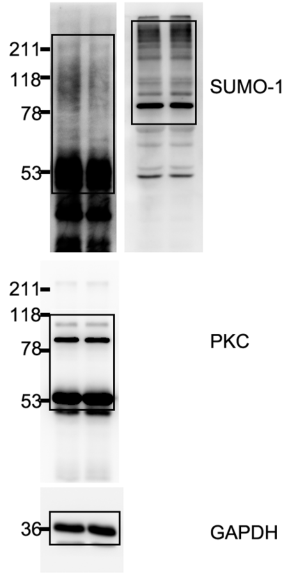

Supplementary Fig. 6d

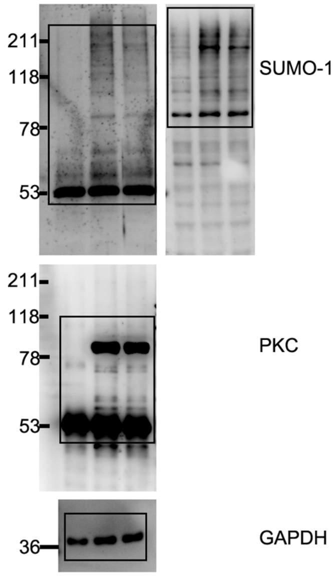

Supplementary Figure 8 continued. Full images of western blots.
